# Individual and partnership characteristics associated with consistent condom use in a cohort of cisgender men who have sex with men and transgender women in Nigeria

**DOI:** 10.1186/s12889-021-11275-w

**Published:** 2021-06-30

**Authors:** Oluwasolape Olawore, Trevor A. Crowell, Sosthenes C. Ketende, Habib O. Ramadhani, Hongjie Liu, Julie A. Ake, Afoke Kokogho, Sylvia Adebajo, Man E. Charurat, Rebecca G. Nowak, Stefan D. Baral, on behalf of the TRUST/RV368 Study Group, Manhattan Charurat, Julie Ake, Aka Abayomi, Sylvia Adebajo, Stefan Baral, Trevor Crowell, Charlotte Gaydos, Afoke Kokogho, Jennifer Malia, Olumide Makanjuola, Nelson Michael, Nicaise Ndembi, Rebecca Nowak, Oluwasolape Olawore, Zahra Parker, Sheila Peel, Habib Ramadhani, Merlin Robb, Cristina Rodriguez-Hart, Eric Sanders-Buell, Elizabeth Shoyemi, Sodsai Tovanabutra, Sandhya Vasan

**Affiliations:** 1 Johns Hopkins Bloomberg School of Public Health, 615 North Wolfe Street, Suite 3507, Baltimore, MD 21205 USA; 2 U.S. Military HIV Research Program, Walter Reed Army Institute of Research, Silver Spring, MD USA; 3 Henry M. Jackson Foundation for the Advancement of Military Medicine, Bethesda, MD USA; 4 Institute of Human Virology, University of Maryland School of Medicine, Baltimore, MD USA; 5 School of Public Health, University of Maryland, College Park, MD USA; 6 HJF Medical Research International, Abuja, Nigeria; 7 U.S. Army Medical Research Directorate – Africa, Nairobi, Kenya; 8 Maryland Global Initiatives Corporation (MGIC), Abuja, Nigeria

**Keywords:** Men who have sex with men, Nigeria, condoms, Networks, HIV, Epidemiology, Sexual and gender minorities

## Abstract

**Background:**

This study reports on the individual and partnership characteristics that influence consistent condom use in cisgender men who have sex with men (MSM) and transgender women (TGW) attending trusted community centers that provide HIV prevention and treatment services in Nigeria.

**Methods:**

Adults assigned male at birth who reported anal sex with male partners who enrolled between March 2013–2019 and had information about at least one male sexual partner were included in these analyses. At enrollment and follow-up visits every 3 months for up to 18 months, participants were administered detailed questionnaires that collected information about demographics, sexual practices, HIV risk behaviors, and characteristics and behaviors of their partners in the previous year (at enrollment) or the preceding 3 to 6-months (at follow-up visits). Logistic regression models with generalized estimating equations were used to assess the odds ratio (OR) and 95% confidence intervals (CI) of individual, partner, and partnership characteristics associated with consistent condom use (CCU). A participant was defined as consistently using condom if they reported always using condoms all the time they had insertive, receptive or both types of anal sex with a male partner.

**Results:**

At the individual level, CCU was positively associated with higher education, disclosure of key population status to a healthcare worker and negatively associated with poor access to condoms. At the partner and partnership level, CCU was associated with partners with higher education (aOR: 1.36; 95% CI: 1.07–1.72), casual relationships (aOR: 1.22; 95% CI: 1.11–1.34) and relationships in which partners encouraged the participant to use condoms with other partners (aOR: 1.14; 95% CI: 1.02–1.28). Relationships in which the partner was married to a woman and/or the partner’s HIV status positive or unknown were negatively associated with CCU.

**Conclusions:**

These findings suggest that individuals in relationships where partners were more open and encouraged safer sex were more likely to consistently use condoms. HIV prevention programs should consider leveraging communication to sexual partners to encourage condom use as this may support condom use with other sexual partners. Given sustained and growing HIV and STI epidemics among MSM and TGW, even with pre-exposure prophylaxis scale-up, it is crucial to continue to study optimal implementation strategies to increase condom use.

**Supplementary Information:**

The online version contains supplementary material available at 10.1186/s12889-021-11275-w.

## Background

Even in the context of a mixed HIV epidemic in Nigeria, cisgender men who have sex with men (MSM) and transgender women (TGW) bear a disproportionate burden of infection with estimated HIV prevalence 18–66% compared to about 4% among reproductive aged adults [1, 21, 42, 43]. This increased burden is due to the high HIV transmission risks associated with condomless anal intercourse with viremic partners [3, 13, 29]. It has been estimated that the 5-year cumulative incidence of HIV among MSM would be reduced by 80–98% if the efficiency of HIV transmission in anal sex was similar to the efficiency of transmission in condomless vaginal sex [3]. While the use of pre-exposure prophylaxis (PrEP) is effective in reducing transmission of HIV, it is not routinely available in many sub-Saharan African (SSA) countries. Thus, consistent condom use (CCU) remains a cost-effective HIV prevention strategy writ large.

Individual-level determinants of CCU among MSM in Nigeria have been shown to include knowledge of HIV transmission, frequency of sex, and alcohol or drug use before or during sex [39]. However, factors influencing condom use may not be fully captured by a single measure and understanding the dynamics between the attributes of the sexual relationship, partner characteristics, and individual level factors is necessary to fully characterize the determinants of condom usage and opportunities for intervention [5, 24, 41].

Among MSM, research has consistently shown unprotected anal intercourse is more likely in committed or more serious relationships compared to relationships that are more casual [7, 15, 27, 40]. This dynamic has been attributed to higher levels of trust and familiarity with committed partners and the perception that condom use may affect intimacy [10, 15, 18, 23, 48]. Perceived and actual partner characteristics have also been shown to influence individual decisions to use condom. For example, MSM at risk for HIV may be more likely to use condom with partners they perceive to be more likely to be living with HIV and serosorting is common among MSM when both partners have disclosed their HIV statuses [27, 35, 47]. Other factors such as age difference between partners, sexual partner concurrency, and partner norms around condoms use may also impact CCU [5, 26, 27].

Although TGW have been shown to have higher odds of reporting condomless receptive anal sex with male partners compared to MSM, few studies have characterized the factors that drive this association in TGW specifically [32]. A study among TGW in China found that subjective norms, condom use attitude, condom use skills, and self-efficacy were associated with frequent condom use and knowledge of HIV was not related to condom use [6, 45]. Other factors that have been shown to be associated with condomless sex among TGW include feminizing medical intervention, factors related to sex-work [6].

While various studies have provided evidence for the partner and relationship characteristics that may influence individual decisions to consistently use condoms, there is limited research on the subject matter among MSM and TGW in sub-Saharan Africa, where cultural factors, resource limitations and other factors may influence the dynamics of condom negotiation and decision-making in ways that differ from those in westernized settings. Given limited condom uptake among cisgender MSM and TGW in Nigeria [8, 39, 44] and the continued importance of characterizing optimal strategies to encourage CCU, we assessed the individual, relationship and partner-level factors associated with CCU among a cohort of cisgender MSM and TGW enrolled in two community-engaged clinics in Abuja and Lagos, Nigeria, thus identifying characteristics that may aid in developing targeted strategies.

## Methods

### Study design and population

TRUST/RV368 study is a prospective cohort study in Lagos and Abuja, Nigeria that assesses the effectiveness of network-based recruitment of MSM and TGW into an HIV prevention and treatment program. As described elsewhere, participants are recruited into the study using respondent-driven sampling; RDS [2, 8, 33]. Briefly, participants are eligible to participate if they were assigned male at birth, are aged ≥16 years (Abuja) or ≥ 18 years (Lagos), and reported receptive or insertive anal intercourse with a male partner in the past year. Initial seed participants were recruited through community-based sampling of individuals who were identified to be well-connected within the population of interest. Each seed was given three RDS coupons to distribute to eligible peers and each individual subsequently recruited was also given three coupons to distribute until equilibrium was achieved.

At enrollment and follow-up visits every 3 months for up to 18 months, participants were administered a detailed questionnaire that collected information about demographics, sexual practices, and HIV risk behaviors in the previous year (at enrollment) or the preceding 3 to 6-months (at follow-up visits). Participants also provided information related to demographics and risk behaviors of up to five of their sexual partners from the past year. At each visit, participants were counselled and screened for HIV and other sexually transmitted infections (STIs). Blood samples were tested for HIV using a parallel testing algorithm of two rapid tests according to national guidelines in Nigeria [28]. The questionnaires used (supplemental file) were developed for the study incorporating modifications of previously validated scales such as the PHQ9 depression module and the World Bank’s Social Capital Tool [22, 46].

Baseline data for participants enrolled in the study between April 2013 and April 2019 who provided information on at least one sexual partner at enrollment were included in these analyses. All participants provided written informed consent in English or Hausa. The study was approved by the University of Maryland Baltimore Institutional Review Board (IRB); the Federal Capital Territory Health Research Ethics Committee, Abuja; and Walter Reed Army Institute of Research IRB.

### Outcomes of interest

The three outcomes of interest were CCU with insertive anal sex, CCU with receptive anal sex, and CCU with both types of anal sex in the past year. For each anal sex type, participants were asked “In the past year, when you had anal sex with [partner name], how often was a condom used?”. A participant was defined as consistently using condom if they reported always using condoms all the time they had insertive, receptive or both types of anal sex with a male partner. Each outcome was examined in a separate model.

### Predictors of interest

#### Participant-level factors

Participant-level factors of interest included demographic characteristics such as age, education status, marital status, sexual orientation and gender identity. Other participant-level variables of interest were disclosure of sexual or gender identity to a healthcare worker, drinking habits in the past 30 days, access to condoms, knowledge of HIV risks, history of STI and HIV testing, previous STI diagnosis, self-reported HIV status, and HIV testing results at baseline.

#### Partner and relationship-level factors

Partner and relationship-level factors were all reported from the perspective of the participant. Partner-level variables included partner’s age, education status, sexual orientation, marital status, sexual concurrency, condom use with their other partners, alcohol or drug use, STI history, and HIV status.

Relationship-level variables included relationship type, age difference between participant and partner socio-economic difference between participant and partner, frequency of sexual intercourse with partner, how often HIV was discussed in relationship, and HIV serostatus concordance based on self-reported HIV status. Sexual network density—based on the answer to the question “Does sexual partner one know sexual partner two?” and so on up to the fifth sexual partner as necessary—was also assessed. Sexual network density was defined as degree of connection between the sexual partners of a participant and was calculated as the total number of actual ties divided by the total number of potential ties. Network density scores were dichotomized with < 50% considered “smaller” and ≥ 50% considered “larger” network density.

### Statistical analyses

Demographic characteristics and sexual behaviors of study participants and at most five of their partners were examined at enrollment using descriptive statistics.

Odds ratios (ORs) and adjusted odds ratios (aORs) with corresponding 95% confidence intervals (CI) of participant and partnership covariates associated with CCU during insertive, receptive, and both types of anal sex were calculated using logistic regression with generalized estimating equations to account for clustering within participants. Variables significant at *p* ≤ 0.10 and pre-specified variables including age, marital status, gender identity, and education status previously known to be associated with CCU were included in the multiple regression analyses regardless of statistical significance. *1*  0.05 was considered significant in the multiple regression analyses. Analyses were carried out as complete case analyses and refusal/don’t know responses were kept as categories the final model where applicable. Sub-group analyses assessing the associated factors in the two KP groups separately i.e., MSM and TGW were also carried. All analyses were performed using Stata (version 15; StataCorp LP, College Station, Texas USA).

## Results

Between April 2013 and April 2019, 2591 eligible MSM and TGW participants were recruited into the study. Of those, 1786 reported on a total of 6432 sexual partners at enrollment and were included in these analyses. The average sex network size was 3.6.

### Participant characteristics

An overview of the demographic and behavioral characteristics of the participants in this study is presented in Table 1. Seventy-four percent (*n* = 1324) of the participants were recruited from Abuja and the median age of the participants was 24 years (interquartile range [IQR]: 21–27). Eighty percent (*n* = 1424) self-identified as male and the median number of sexual partners reported in the past year was 4 (IQR: 2–8). Five hundred and thirty (30%) participants reported being gay or homosexual while 70% reported being bisexual (*n* = 1238). Most of the participants had a secondary education or higher and majority of them reported being single or never married.

**Table 1:** Characteristics of men who have sex with men and transgender women at enrollment into the TRUST/RV368 cohort in Abuja and Lagos, Nigeria

Participant Characteristics (***N*** = 1786)	n (%)
**Site**	
Abuja	1324 (74.1)
Lagos	462 (25.9)
**Age categories**	
16–19 years	255 (14.3)
20–24 years	752 (42.1)
25–29 years	503 (28.2)
30–34 years	171 (9.6)
35 and older	94 (5.3)
Missing	11 (0.6)
**Sexual orientation**	
Gay or homosexual	530 (29.7)
Bisexual	1238 (69.3)
Other	2 (0.1)
Refusal/don’t know /missing	16 (0.9)
**Gender identity**	
Male	1424 (79.7)
Female	180 (10.1)
Both/Other	163 (9.1)
Refusal/don’t know/missing	19 (1.1)
**Education level**	
None/Primary education only	87 (4.9)
Junior secondary only	96 (5.4)
Senior secondary	920 (51.5)
Higher than senior secondary	661 (37.0)
Refusal/don’t know/missing	22 (1.2)
**Marital status**	
Single/never married	1612 (90.3)
Married/cohabitating with a woman	92 (5.2)
Cohabitating with a man	23 (1.3)
Divorced/separated/widowed/other	44 (2.5)
Refusal/don’t know/missing	15 (0.84)
**Ever disclosed MSM status to healthcare worker**	
No	1204 (67.4)
Yes	565 (31.6)
Refusal/don’t know	5 (0.3)
Missing	12 (0.7)
**Access to condoms**	
Gets condoms for free	531 (29.7)
Buys condoms	683 (38.2)
Gets for free and buys them	502 (28.1)
Neither buys condom nor gets them for free	43 (2.4)
Refusal/don’t know/missing	27 (1.5)
**Episodes of heavy drinking in the past 30 days**	143 (8.0)
**Social Cohesion**	
Low social cohesion	911 (51.0)
Medium social cohesion	347 (19.4)
High social cohesion	528 (29.6)
**Knowledge of HIV risks**	
a.) What type of sex is associated with highest HIV transmission risk?	
All types of sex carry equal risk	1027 (57.5)
Anal sex	384 (21.5)
Vaginal sex	237 (13.3)
Oral	60 (3.4)
Refusal/Don’t know	62 (3.5)
Missing	16 (0.9)
b.) What type of anal sex is associated with the highest HIV transmission risk?	
All types of anal sex carry equal risk	784 (43.9)
Receptive anal sex	735 (41.2)
Insertive anal sex	169 (9.5)
Refusal/don’t know	78 (4.4)
Missing	20 (1.1)
**Any STI test in the past 12 months**	
No	1179 (66.0)
Yes	592 (33.2)
Refusal/don’t know	3 (0.2)
Missing	12 (0.7)
**Any STI diagnosis in the past 12 months**	
No	1358 (76.0)
Yes	388 (21.7)
Refusal/don’t know	9 (0.5)
Missing	31 (1.7)
**How worried have you been about HIV in the past 12 months?**	
Not at all worried	846 (47.4)
Somewhat/ a little worried	468 (26.2)
Very worried	452 (25.3)
Refusal/don’t know/missing	20 (1.1)
**Ever previously tested for HIV?**	
No	358 (20.0)
Yes, once	422 (23.6)
Yes, more than once	989 (55.4)
Refusal/don’t know/missing	17 (1.0)
**Self-reported HIV status**	
Negative	883 (49.4)
Positive	487 (27.3)
Unknown	416 (23.3)
**HIV rapid test results**	
Negative	936 (52.4)
Positive	842 (47.1)
Missing	8 (0.5)
**Sexual network density**	
Smaller network density	561 (31.4)
Larger network density	956 (53.5)
Unknown network density	269 (15.1)

Ninety-six percent (*n* = 1715) of participants reported either buying condoms or getting them for free. A majority of the participants reported that all types of sex were associated with equal risk of HIV transmission, while 22% (*n* = 384) reported anal sex being the type of sex associated with the highest risk of transmission. Seventy-nine percent (*n* = 1411) of the participants had ever been previously tested for HIV and a quarter of the participants reported being very worried about HIV in the past 12 months Of the 1786 participants included in these analyses, 47% (*n* = 842) were living with HIV.

### Partner and relationship characteristics

The participant reported characteristics of the partners and relationships are presented in Table 2. The median age of partners reported on was 27 (IQR: 23–32) and 65% (*n* = 4106) of the partners were reported to be casual partners. Most of the partners (*n* = 5285; 82%) were single or never married and 53% (*n* = 3418) were reported to be bisexual. A majority of the partners had a senior secondary education or higher and more than half of the partners reported on had a socio-economic status that was higher than the participants’.

**Table 2:** Partner and partnership characteristics by CCU with both types of anal sex

Partner and relationship characteristics	Total (***N*** = 6432)	CCU with both types of anal sex (***N*** = 2853) (44.3%)	Non-CCU with both types of anal sex (***N*** = 3579) (55.7%)	***p*** value
**Partner age categories**				
Younger than 25	2225 (34.6)	1047 (36.7)	1178 (32.9)	
25–34	2904 (45.2)	1324 (46.4)	1580 (44.2)	
35–44	546 (8.5)	209 (7.3)	337 (9.4)	< 0.001
45 and older	134 (2.1)	47 (1.7)	87 (2.4)	
Refusal/Don’t know	623 (9.7)	226 (7.9)	397 (11.1)	
**Partner’s education status**				
None/Primary education only	168 (2.6)	50 (1.8)	118 (3.3)	
Junior secondary only	127 (2.0)	49 (1.7)	78 (2.2)	< 0.001
Senior secondary	2107 (32.8)	943 (33.1)	1164 (32.5)	
Higher than senior secondary	3349 (52.1)	1574 (55.2)	1775 (49.6)	
Refusal/Don’t know	681 (10.6)	237 (8.3)	444 (12.4)	
**Partner’s sexual orientation**				
Gay or homosexual	2487 (38.7)	1131 (39.7)	1356 (37.9)	
Bisexual	3418 (53.1)	1532 (53.7)	1886 (52.7)	< 0.001
Refusal/Don’t know	527 (8.2)	190 (6.7)	337 (9.4)	
**Partner’s marital status**				
Single/Never married	5285 (82.2)	2338 (82.0)	2947 (82.3)	
Married to a woman	919 (14.3)	397 (13.9)	522 (14.6)	
Cohabitating with a man	20 (0.3)	7 (0.3)	13 (0.4)	0.033
Separated/Divorced/Widowed	39 (0.6)	17 (0.6)	22 (0.6)	
Refusal/Don’t know	116 (1.8)	69 (2.4)	47 (1.3)	
Missing	53 (0.8)	25 (0.88)	28 (0.80)	
**Relationship type**				
Regular	2255 (35.1)	951 (33.3)	1304 (36.4)	
Casual	4160 (64.7)	1890 (66.3)	2270 (63.4)	0.011
Refusal/Don’t know	8 (0.12)	6 (0.2)	2 (0.06)	
Missing	9 (0.14)	6 (0.2)	3 (0.08)	
**Age difference between partner and participant**				
No age difference	548 (8.5)	237 (8.3)	311 (8.7)	
Age difference greater than or equal to ten years	998 (15.5)	440 (15.4)	558 (15.6)	< 0.001
Age difference less than 10 years	4229 (65.8)	1938 (67.9)	2291 (64.0)	
Unknown age difference	657 (10.2)	238 (8.3)	419 (11.7)	
**Partner Age mix**				
All partner’s ages were different	3264 (50.8)	1470 (51.5)	1794 (50.1)	
All partners were consistently younger than participant	755 (11.7)	354 (12.4)	401 (11.2)	
All partners were consistently older than participant	1976 (30.7)	894 (31.3)	1082 (30.2)	< 0.001
All partners were the same age as participant	42 (0.7)	16 (0.6)	26 (0.7)	
Unknown age mix	395 (6.1)	119 (4.2)	276 (7.7)	
**Partner socio-economic status**				
Same as participant’s	1367 (21.3)	635 (22.3)	732 (20.5)	
Lower than participant’s	1386 (21.6)	610 (21.4)	776 (21.7)	
Higher than participant’s	3428 (53.3)	1520 (53.3)	1908 (53.3)	0.012
Refusal/Don’t know	248 (3.9)	88 (3.1)	160 (4.5)	
Missing	3 (0.05)	0	3 (0.08)	
**Did your partner have more than one regular sexual partner at the same time?**				
No	963 (15.0)	448 (15.7)	515 (14.4)	
Yes	4403 (68.5)	1930 (67.7)	2473 (69.1)	0.311
Don’t Know	1066 (16.6)	475 (16.6)	591 (16.5)	
**How many sexual partners do you believe your partner has had in the past year?**				
No sexual partners	104 (1.6)	44 (1.5)	60 (1.7)	
1–2 sexual partners	470 (7.3)	244 (8.6)	226 (6.3)	
3–5 sexual partners	573 (8.9)	310 (10.9)	263 (7.4)	< 0.001
Greater than 5	589 (9.2)	309 (10.9)	280 (7.8)	
Don’t know	4696 (73.0)	1946 (68.2)	2750 (76.8)	
**Do you believe partner has ever had sex under the influence of drug or alcohol?**				
No	2100 (32.7)	1031 (36.1)	1069 (29.9)	
Yes	1238 (19.3)	623 (21.8)	615 (17.2)	
Don’t Know	778 (12.1)	344 (12.1)	434 (12.1)	
Refusal	2316 (36.0)	855 (30.0)	1461 (40.8)	
**When partner has sex with other sexual partners how often do you believe a condom is used**				
Never	237 (3.7)	82 (2.9)	155 (4.3)	
Sometimes	1035 (16.1)	446 (15.6)	589 (16.5)	
Almost always	296 (4.6)	124 (4.4)	172 (4.8)	< 0.001
Always	974 (15.1)	657 (23.0)	317 (8.9)	
Don’t know	1311 (20.4)	541 (19.0)	770 (21.5)	
Refusal	2577 (40.1)	1001 (35.1)	1576 (44.0)	
Missing	2 (0.03)	2 (0.07)	0	
**When you had sex with this partner, how often did you have sex?**				
Only once or twice ever	1125 (17.5)	564 (19.8)	561 (15.7)	
Almost every day	207 (3.2)	80 (2.8)	127 (3.6)	
A few times each week	1003 (15.6)	411 (14.4)	592 (16.5)	
A few times each month	1478 (23.0)	651 (22.8)	827 (23.1)	< 0.001
Once a month	2598 (40.4)	1140 (40.0)	1458 (40.7)	
Refusal/Don’t know	20 (0.3)	6 (0.2)	14 (0.4)	
Missing	1 (0.02)	1 (0.04)	0	
**As far as you know, has partner ever had any kind of STI?**				
No	2479 (38.5)	1256 (44.0)	1223 (34.2)	
Yes	551 (8.6)	233 (8.2)	318 (8.9)	< 0.001
Unknown	3371 (52.4)	1355 (47.5)	2016 (56.3)	
Missing	31 (0.5)	9 (0.3)	22 (0.6)	
**Has partner encouraged you to make sure you use condom when you have sex with other sexual partners?**				
No	2274 (35.4)	826 (29.0)	1, 448 (40.5)	
Yes	3343 (52.0)	1661 (58.2)	1682 (47.0)	
Don’t know	11 (0.2)	0 (0.0)	11 (0.3)	< 0.001
Refusal	804 (12.5)	366 (12.9)	438 (12.2)	
**How frequently do you discuss HIV with partner?**				
Never	2059 (32.0)	756 (26.5)	1303 (36.4)	
At least once a week	1173 (18.2)	668 (23.4)	505 (14.1)	
Once a month or less	2395 (37.2)	1057 (37.1)	1338 (37.4)	< 0.001
Refused	805 (12.5)	372 (13.0)	433 (12.1)	
**As far as you know what your partner’s HIV status is?**				
Negative	2598 (40.4)	1931 (48.8)	1207 (33.7)	
Positive	355 (5.5)	127 (4.5)	228 (6.4)	
Unknown	3460 (53.8)	1330 (46.6)	2130 (59.5)	< 0.001
Missing	19 (0.3)	5 (0.2)	14 (0.4)	
**HIV serostatus concordance**				
Both negative	1363 (21.2)	730 (25.6)	633 (17.7)	
Both positive	243 (3.8)	94 (3.3)	149 (4.2)	
HIV serodiscordant/ Unknown status	3972 (61.8)	1784 (62.5)	2188 (61.1)	< 0.001
Both unknown	835 (13.0)	240 (8.4)	595 (16.6)	
Missing	19 (0.3)	23 (0.4)	14 (0.4)	

Sixty-eight percent of the partners reported having multiple regular sexual partners at the same time, while only 15% of the partners were believed to always consistently use condoms when they had had sex with their other sexual partners. Condom use was encouraged by 52% of the partners and 18% of the partners discussed HIV with the participant at least once a week.

The HIV status of 54% of the partners were unknown by the participants while 6% of the partners were reported to be HIV positive. Using the self-reported HIV status of the participants and the participant reported HIV status of the partners, 4% of the partnerships were HIV positive seroconcordant and 75% of the partnership were HIV-serodiscordant or had unknown seroconcordance status.

### Individual characteristics associated with consistent condom use

The bivariate and multivariable analyses of the participant characteristics associated with CCU with insertive, receptive, both types of anal sex are presented in Table 3. The results of the analysis of CCU with both types of anal sex are reported here.

**Table 3:** Participant characteristics associated with consistent condom use with anal sex

Participant characteristics	Associations with insertive anal sex		Associations with receptive anal sex		Associations with both types of anal sex	
	Bivariate analyses (95% CI)	Multivariable analyses (95% CI)	Bivariate analyses (95% CI)	Multivariable analyses (95% CI)	Bivariate analyses (95% CI)	Multivariable analyses (95% CI)
**Age categories**						
16–19 years	Ref	Ref	Ref	Ref	Ref	Ref
20–24 years	0.71 (0.50–0.99) **	0.55 (0.38–0.81) **	0.94 (0.71–1.24)	0.76 (0.56–1.03)	0.89 (0.70–1.15)	0.75 (0.57–0.99) **
25–29 years	0.61 (0.43–0.88) **	0.41 (0.28–0.62) ***	0.88 (0.65–1.18)	0.57 (0.41–0.80) **	0.79 (0.60–1.03) *	0.58 (0.43–0.78) **
30–34 years	0.72 (0.47–1.11)	0.51 (0.31–0.83) **	0.91 (0.60–1.37)	0.61 (0.38–0.97) **	0.86 (0.61–1.22)	0.64 (0.43–0.95) **
35 and older	0.71 (0.42–1.19)	0.52 (0.28–0.97) **	0.92 (0.51–1.62)	0.62 (0.31–1.24)	0.92 (0.59–1.43)	0.72 (0.43–1.21)
**Education status**						
None/primary education only	Ref	Ref	Ref	Ref	Ref	Ref
Junior secondary only	0.87 (0.44–1.71)	0.76 (0.36–1.60)	1.15 (0.61–2.12)	1.03 (0.52–2.05)	1.32 (0.75–2.33)	1.25 (0.68–2.28)
Senior secondary	1.98 (1.20–3.27) **	1.50 (0.84–2.58)	1.48 (0.90–2.44)	1.13 (0.64–1.98)	1.82 (1.17–2.82) **	1.42 (0.87–2.31)
Higher than secondary	2.02 (1.22–3.34) **	1.78 (1.01–3.14) **	1.94 (1.16–3.23) **	1.66 (0.94–2.94)	2.05 (1.31–3.21) **	1.80 (1.10–2.94) **
Refusal/Don’t know	1.42 (0.36–5.51)	1.76 (0.37–8.4)	1.24 (0.27–5.69)	1.42 (0.26–7.74)	1.24 (0.33–4.60)	1.47 (0.35–6.15)
**Sexual orientation**						
Gay or homosexual	Ref		Ref		Ref	
Bisexual	1.17 (0.93–1.48)		1.04 (0.85–1.27)		1.09 (0.91–1.30)	
Other	–		1.94 (0.19–19.7)		1.98 (0.20–20.0)	
Refusal/Don’t know	4.02 (0.64–25.5)		1.38 (0.09–22.2)		2.95 (0.42–20.8)	
**Gender identity**						
Male	Ref	Ref	Ref	Ref	Ref	Ref
Female	1.45 (0.97–2.17) *	1.52 (0.97–2.36)	1.15 (0.87–1.52)	1.21 (0.90–1.63)	1.19 (0.92–1.55)	1.21 (0.91–1.60)
Both/other	0.82 (0.57–1.17)	0.81 (0.56–1.18)	0.68 (0.49–0.96) **	0.69 (0.48–0.98) **	0.76 (0.56–1.03) *	0.77 (0.56–1.06)
Refusal/Don’t know	0.82 (0.14–4.71)	0.72 (0.09–5.47)	0.30 (0.03–2.70)	0.25 (0.03–2.20)	0.47 (0.09–2.50)	0.43 (0.07–2.59)
**Marital status**						
Single/Never Married	Ref	Ref	Ref	Ref	Ref	Ref
Married/Cohabitating with a woman	0.99 (0.64–1.52)	1.39 (0.84–2.32)	1.05 (0.66–1.68)	1.28 (0.75–2.17)	0.99 (0.66–1.47)	1.27 (0.81–2.00)
Cohabitating with a man	1.31 (0.63–2.74)	0.78 (0.35–1.73)	0.89 (0.45–1.76)	0.62 (0.32–1.23)	1.05 (0.58–1.89)	0.72 (0.39–1.33)
Divorced/Separated/Widowed	0.70 (0.36–1.37)	0.67 (0.31–1.41)	1.11 (0.62–1.96)	1.10 (0.58–2.07)	0.92 (0.54–1.56)	0.86 (0.49–1.52)
**Disclosure to healthcare worker**						
No	Ref	Ref	Ref	Ref	Ref	Ref
Yes	1.65 (1.33–2.05) ^***^	1.31 (1.03–1.66) **	1.67 (1.36–2.05) ***	1.39 (1.11–1.76) **	1.47 (1.24–1.75) ***	1.22 (1.00–1.48) **
Refusal/Don’t know	0.83 (0.15–4.71)	1.09 (0.15–7.7)	0.13 (0.02–0.77) **	0.09 (0.02–0.43) **	0.45 (0.07–2.72)	0.46 (0.06–3.32)
**Heavy Drinking in past 30 days**						
No	Ref		Ref		Ref	
Yes	1.20 (0.85–1.69)		1.12 (0.77–1.64)		1.12 (0.82–1.52)	
Refusal/Don’t know	0.72 (0.36–1.45)		0.81 (0.38–1.76)		0.75 (0.40–1.38)	
**Access to condoms**						
Gets condoms for free	Ref	Ref	Ref	Ref	Ref	Ref
Buys condoms	0.69 (0.54–0.88) **	0.82 (0.63 1.07)	0.74 (0.58–0.94) **	0.91 (0.70–1.17)	0.77 (0.62–0.94) **	0.91 (0.73–1.13)
Gets for free and buys them	0.87 (0.67–1.13)	0.81 (0.61–1.06)	0.90 (0.70–1.15)	0.92 (0.71–1.20)	0.88 (0.71–1.09)	0.86 (0.68–1.07)
Neither	0.14 (0.04–0.44) **	0.23 (0.06–0.81) **	0.15 (0.06–0.38) ***	0.18 (0.07–0.47) ***	0.19 (0.09–0.43) ***	0.26 (0.11–0.59) **
Refusal/Don’t know	0.81 (0.22–3.03)	1.53 (0.32–7.24)	0.89 (0.30–2.70)	1.32 (0.39–4.47)	0.72 (0.25–2.06)	1.18 (0.39–3.59)
**Knowledge of HIV risks**						
a.) What type of sex is associated with highest HIV transmission risk?						
All types of sex carry equal risk	Ref	Ref	Ref	Ref	Ref	Ref
Anal sex	1.32 (1.02–1.70) **	1.15 (0.88–1.50)	1.35 (1.07–1.70) **	1.19 (0.93–1.52)	1.31 (1.07–1.60) **	1.16 (0.94–1.44)
Vaginal sex	1.15 (0.85–1.57)	1.28 (0.92–1.80)	1.22 (0.87–1.70)	1.47 (1.03–2.10) **	1.12 (0.86–1.46)	1.26 (0.95–1.68)
Oral	1.06 (0.61–1.87)	1.31 (0.72–2.40)	1.65 (0.98–2.77) *	1.78 (1.04–3.04) **	1.36 (0.86–2.15)	1.48 (0.92–2.37)
Refusal/Don’t know	0.76 (0.42–1.39)	1.20 (0.63–2.27)	0.82 (0.47–1.44)	1.11 (0.61–2.03)	0.75 (0.45–1.23)	1.04 (0.61–1.76)
b.) What type of anal sex is associated with the highest HIV transmission risk?						
All types of anal sex carry equal risk	Ref		Ref		Ref	
Receptive anal sex	1.01 (0.81–1.25)		1.14 (0.93–1.40)		1.08 (0.90–1.29)	
Insertive anal sex	1.02 (0.72–1.46)		1.19 (0.82–1.75)		1.15 (0.85–1.56)	
Refusal/Don’t know	0.85 (0.52–1.40)		0.79 (0.45–1.40)		0.87 (0.56–1.35)	
**Any STI test in the past 12 months**						
No	Ref	Ref	Ref	Ref	Ref	Ref
Yes	1.33 (1.08–1.64) **	1.18 (0.94–1.48)	1.39 (1.13–1.70) **	1.24 (0.99–1.55)	1.28 (1.07–1.52) **	1.14 (0.94–1.37)
Refusal/Don’t know	6.28 (0.94–41.8)	5.23 (0.45–60.3)	2.33 (0.33–16.3)	2.28 (0.25–20.6)	6.15 (0.94–40.4)	6.00 (0.68–53.2)
**Any STI diagnosis in the past 12 months**						
No	Ref		Ref		Ref	
Yes	0.93 (0.76–1.18)		0.84 (0.67–1.06)		0.88 (0.72–1.07)	
Refusal/ Don’t know	0.53 (0.13–2.09)		0.43 (0.08–2.34)		0.52 (0.15–1.81)	
**Worry about HIV in the past 12 months?**						
Not at all worried	Ref	Ref	Ref	Ref	Ref	Ref
Somewhat/ a little worried	0.94 (0.74–1.20)	0.83 (0.70–1.08)	1.06 (0.84–1.33)	0.95 (0.74–1.21)	0.97 (0.79–1.18)	0.87 (0.70–1.07)
Very worried	0.74 (0.58–0.94) **	0.65 (0.50–0.84) **	0.75 (0.59–0.95) **	0.65 (0.51–0.84) **	0.75 (0.61–0.92) **	0.66 (0.53–0.82) **
Refusal/Don’t know	0.55 (0.47–0.63) ^***^	0.65(0.44–0.97) **	–	–	1.89 (0.27–13.2)	2.27 (0.27–18.7)
**Ever previously tested for HIV?**						
No	Ref	Ref	Ref	Ref	Ref	
Yes, once	1.53 (1.11–2.10) **	1.07 (0.56–2.06)	1.59 (1.19–2.13) **	1.89 (1.01–3.56) **	1.51 (1.17–1.97) **	1.44 (0.86–2.43)
Yes, more than once	1.98 (1.50–2.61) ***	1.36 (0.70–2.67)	1.78 (1.37–2.30) ***	2.02 (1.05–3.89) **	1.77 (1.41–2.22) ***	1.63 (0.95–2.80)
Refusal/Don’t know	13.9 (2.79–68.9) **	22.1 (6.68–67.5)	5.75 (4.60–7.18) ***	9.72 (4.93–19.2) ***	13.2 (2.68–65.4) **	20.0 (6.6–60.3) **
**Self-reported HIV**						
Negative	Ref	Ref	Ref	Ref	Ref	Ref
Positive	1.30 (1.03–1.65) **	1.18 (0.91–1.54)	1.29 (1.04–1.61) **	1.20 (0.94–1.54)	1.22 (1.01–1.48) **	1.13 (0.91–1.39)
Unknown	0.62 (0.48–0.81) ***	0.82 (0.44–1.54)	0.72 (0.56–0.92) **	1.53 (0.83–2.84)	0.68 (0.55–0.85) **	1.10 (0.67–1.82)
**HIV results at baseline**						
Negative	Ref		Ref		Ref	
Positive	0.90 (0.73–1.10)		0.89 (0.73–1.08)		0.90 (0.76–1.06)	
**Site**						
Abuja	Ref	Ref	Ref	Ref	Ref	Ref
Lagos	2.03 (1.62–2.54) ***	1.76 (1.36–2.29) ***	1.47 (1.21–1.79) ***	1.21 (0.95–1.54)	1.60 (1.34–1.89) ***	1.41 (1.15–1.73) **

* *p* ≤ 0.10

** *p* < 0.05

*** *p* < 0.001

In the bivariate analysis, higher education status disclosure of MSM status to a healthcare worker, previous STI and HIV tests, self-reported positive HIV status, and knowledge that anal sex had the highest HIV transmission risk were all positively associated with CCU.

Age between 20 and 24, participants who identified as women, buying condoms or neither buying nor getting condoms for free, being very worried about HIV in the previous 12 months, and a self -reported positive HIV status were all negatively associated with CCU.

In the multivariable analysis (Table 3), higher education status (aOR: 1.80; 95% CI: 1.10–2.94) and disclosure of MSM status to a healthcare worker (aOR: 1.22 1.00–1.48) remained positively associated with CCU. All age categories except 35 years or older were negatively associated with CCU and so was neither buying nor getting condoms for free (aOR: 0.26; 95% CI: 0.11–0.59), and being very worried about HIV in the previous 12 months (aOR: 0.66; 95% CI: 0.53–0.82).

Results for the sub-group analyses are presented as supplemental tables in the appendix. In the multivariable analysis among TGW (Supplemental Table 1), participants who reported being previously married or widowed and participants who reported that they had previously tested for HIV more than once were more likely to consistently use condoms with both types of anal sex. Conversely, transgender participants who reported their sexual orientation as other were less likely to consistently use condoms with both types of anal sex.

Findings for MSM only (Supplemental Table 3) were similar to results in for the combined analysis. In the multivariable analysis among MSM only, higher education and participants who reported that oral sex had the highest HIV transmission risk were more likely to consistently use condom while younger age group (16–24), neither buying or getting condoms for free, and MSM participants who reported being very worried about HIV in the previous 12 months were less likely to consistently use condoms with both types of anal sex.

### Partner and relationship characteristics associated with consistent condom use

In the multivariable analysis (Table 4), relationships in which the partners had a higher education, casual relationships, relationships where the partner encouraged the participant to use condoms and where HIV was discussed at least once a week and larger network sizes were found to be positively associated with CCU. Older partners (35 and older), relationships where the partner was reported to be married to a woman, all frequencies of sexual acts, and relationships where the partner’s HIV status were reported to be positive, or unknown were all negatively associated with CCU.

**Table 4:** Partner and relationship characteristics associated with consistent condom use with anal sex

Partner and relationship characteristics	Associations with insertive anal sex		Associations with receptive anal sex		Associations with both types of anal sex	
	Bivariate analyses (95% CI)	Multivariable analyses (95% CI)	Bivariate analyses (95% CI)	Multivariable analyses (95% CI)	Bivariate analyses (95% CI)	Multivariable analyses (95% CI)
**Partner age categories**						
Younger than 25	Ref	Ref	Ref	Ref	Ref	Ref
25–34	0.96 (0.87–1.05)	0.93 (0.84–1.03)	1.03 (0.92–1.14)	0.94 (0.84–1.06)	1.00 (0.92–1.09)	0.95 (0.87–1.05)
35–44	0.92 (0.79–1.07)	0.87 (0.72–1.05)	0.98 (0.82–1.16)	0.84 (0.68–1.03)	0.91 (0.79–1.06)	0.84 (0.70–0.99) **
45 and older	0.94 (0.72–1.22)	0.80 (0.59–1.08)	0.77 (0.57–1.05) *	0.62 (0.42–0.90) **	0.81 (0.61–1.07)	0.69 (0.50–0.96) **
Refusal/Don’t know	0.97 (0.78–1.21)	0.27 (0.05–1.47)	0.85 (0.68–1.06)	0.59 (0.16–2.24)	0.89 (0.74–1.07)	0.56 (0.17–1.90)
**Partner’s education status**						
None/Primary education only	Ref	Ref	Ref	Ref	Ref	Ref
Junior secondary only	0.96 (0.72–1.28)	1.01 (0.75–1.36)	0.91 (0.64–1.29)	0.85 (0.59–1.22)	0.91 (0.67–1.25)	0.91 (0.66–1.25)
Senior secondary	1.15 (0.89–1.49)	1.21 (0.92–1.59)	1.24 (0.96–1.59) *	1.27 (0.98–1.65)	1.19 (0.95–1.48)	1.24 (0.99–1.57)
Higher than senior secondary	1.10 (0.85–1.43)	1.22 (0.93–1.59)	1.43 (1.10–1.85) **	1.48 (1.13–1.94) **	1.25 (1.00–1.57) **	1.36 (1.07–1.72) **
Refusal/Don’t know	1.18 (0.86–1.62)	1.28 (0.93–1.78)	1.24 (0.91–1.69)	1.33 (0.97–1.83)	1.19 (0.91–1.56)	1.28 (0.97–1.70)
**Partner’s sexual orientation**						
Gay or homosexual	Ref		Ref		Ref	
Bisexual	0.99 (0.90–1.08)		1.07 (0.96–1.19)		0.99 (0.90–1.08)	
Refusal/Don’t know	1.02 (0.84–1.25)		0.93 (0.72–1.21)		0.91 (0.74–1.12)	
**Partner’s marital status**						
Single/never married	Ref	Ref	Ref	Ref	Ref	Ref
Married to a woman	1.01 (0.92–1.12)	1.04 (0.92–1.18)	0.96 (0.85–1.10)	0.91 (0.78–1.05)	0.95 (0.86–1.06)	0.95 (0.84–1.08)
Cohabitating with a man	0.61 (0.13–2.83)	0.61 (0.15–2.53)	0.32 (0.08–1.19) *	0.33 (0.11–0.98) **	0.32 (0.09–1.11) *	0.33 (0.11–0.97) **
Separated/divorced/widowed	1.02 (0.74–1.41)	1.08 (0.78–1.50)	0.95 (0.60–1.51)	0.93 (0.58–1.48)	1.06 (0.69–1.65)	1.09 (0.71–1.68)
Refusal/Don’t know	1.29 (0.96–1.75) *	1.50 (1.09–2.07) **	1.14 (0.79–1.63)	1.38 (0.93–2.03)	1.34 (0.98–1.82) *	1.66 (1.19–2.33) **
**Relationship type**						
Regular	Ref	Ref	Ref	Ref	Ref	Ref
Casual	1.23 (1.13–1.33) ***	1.13 (1.02–1.24) **	1.29 (1.18–1.41) ***	1.23 (1.10–1.38) ***	1.31 (1.21–1.41) ***	1.22 (1.11–1.34) ***
Refusal/Don’t know	0.55 (0.08–3.93)	0.52 (0.07–3.68)	1.29 (0.74–2.27)	0.98 (0.71–1.34)	0.75 (0.11–5.12)	0.69 (0.08–5.90)
**Age Difference between partner and participant**						
No age difference	Ref	Ref	Ref	Ref	Ref	Ref
Greater than or equal to ten years	0.97 (0.85–1.12)	1.01 (0.86–1.18)	1.11 (0.94–1.31)	1.24 (1.02–1.51) **	1.02 (0.89–1.18)	1.11 (0.95–1.31)
Less than ten years	0.95 (0.85–1.05)	0.95 (0.85–1.06)	1.06 (0.93–1.21)	1.08 (0.93–1.25)	1.00 (0.90–1.11)	1.00 (0.90–1.13)
Unknown age difference	0.96 (0.75–1.21)	3.69 (0.67–20.3)	0.89 (0.70–1.14)	1.63 (0.42–6.35)	0.90 (0.74–1.10)	1.70 (0.49–5.88)
**Partner Age mix**						
All partner’s ages were different	Ref	Ref	Ref	Ref	Ref	Ref
All partners were consistently younger than participant	0.99 (0.75–1.31)	1.05 (0.78–1.40)	0.87 (0.60–1.25)	0.85 (0.58–1.23)	1.05 (0.82–1.36)	1.06 (0.81–1.38)
All partners were consistently older than participant	1.01 (0.80–1.28)	1.11 (0.87–1.42)	0.96 (0.78–1.19)	0.92 (0.74–1.15)	0.99 (0.82–1.19)	0.99 (0.82–1.20)
All partners were the same age as participant	0.66 (0.24–1.83)	0.66 (0.24–1.80)	2.15 (0.78–5.88)	2.54 (0.93–6.9)	1.05 (0.47–2.32)	1.09 (0.49–2.42)
Unknown age mix	0.53 (0.33–0.85) **	0.57 (0.32–1.02)	0.50 (0.31–0.79) **	0.59 (0.33–1.03)	0.56 (0.38–0.84) **	0.63 (0.39–1.03)
**Partner socio-economic status**						
Same as participant’s	Ref	Ref	Ref	Ref	Ref	Ref
Lower than participant’s	1.01 (0.88–1.15)	0.98 (0.85–1.14)	0.97 (0.83–1.14)	0.96 (0.81–1.15)	1.02 (0.89–1.16)	0.99 (0.83–1.11)
Higher than participant’s	0.95 (0.84–1.07)	0.96 (0.84–1.09)	1.08 (0.93–1.25)	1.09 (0.94–1.28)	1.00 (0.89–1.13)	1.02 (0.90–1.16)
Refusal/don’t know	1.00 (0.71–1.42)	1.09 (0.76–1.57)	0.71 (0.51–1.00) *	0.85 (0.59–1.23)	0.90 (0.67–1.22)	0.99 (0.72–1.37)
**Did your partner have more than one regular sexual partner at the same time?**						
No	Ref		Ref		Ref	
Yes	1.14 (0.96–1.36)		1.07 (0.91–1.26)		1.09 (0.96–1.25)	
Don’t know	1.16 (0.89–1.51)		1.16 (0.90–1.50)		1.10 (0.88–1.36)	
**How many sexual partners do you believe your partner has had in the past year?**						
No other sexual partners	Ref	Ref	Ref	Ref	Ref	Ref
1–2 sexual partners	1.11 (0.74–1.66)	1.10 (0.74–1.63)	1.39 (0.95–2.03) *	1.32 (0.90–1.93)	1.24 (0.86–1.80)	1.19 (0.81–1.73)
3–5 sexual partners	1.40 (0.97–2.02) *	1.43 (1.00–2.06)	1.61 (1.13–2.30) **	1.54 (1.08–2.19) **	1.43 (1.00–2.03) *	1.37 (0.96–1.96)
Greater than 5	1.63 (1.09–2.43) **	1.66 (1.11–2.49) **	1.36 (0.92–1.99)	1.32 (0.89–1.96)	1.36 (0.04–1.98)	1.34 (0.91–1.98)
Don’t know	1.28 (0.90–1.83)	1.49 (1.04–2.14) **	1.32 (0.93–1.87)	1.45 (1.01–2.09) **	1.27 (0.90–1.79)	1.37 (0.95–1.96)
**Do you believe partner has ever had sex under the influence of drug or alcohol?**						
No	Ref	Ref	Ref	Ref	Ref	Ref
Yes	1.08 (0.92–1.28)	1.06 (0.89–1.26)	0.91 (0.78–1.06)	0.92 (0.78–1.08)	0.96 (0.84–1.11)	0.96 (0.83–1.11)
Don’t know	0.94 (0.71–1.25)	0.90 (0.67–1.20)	0.87 (0.67–1.13)	0.89 (0.67–1.18)	0.88 (0.70–1.12)	0.87 (0.68–1.11)
Refusal	0.59 (0.47–0.73) ***	0.45 (0.29–0.71) ***	0.60 (0.48–0.74) ***	0.56 (0.43–0.75) ***	0.65 (0.54–0.78) ***	0.60 (0.41–0.89) **
**When partner has sex with other sexual partners how often do you believe a condom is used**						
Never	Ref	Ref	Ref		Ref	Ref
Sometimes	0.73 (0.48–1.10)	0.63 (0.42–0.95) **	1.05 (0.74–1.48)		1.05 (0.78–1.41)	0.94 (0.70–1.27)
Almost always	0.85 (0.52–1.38)	0.73 (0.44–1.21)	0.81 (0.53–1.23)		0.89 (0.62–1.28)	0.82 (0.57–1.17)
Always	0.98 (0.65–1.46)	0.88 (0.59–1.32)	1.22 (0.87–1.71)		1.29 (0.97–1.73) *	1.17 (0.87–1.57)
Don’t know	0.86 (0.56–1.31)	0.82 (0.53–1.27)	0.99 (0.68–1.44)		1.08 (0.79–1.49)	1.12 (0.81–1.55)
Refusal	0.70 (0.47–1.03) *	0.95 (0.58–1.55)	0.79 (0.55–1.15)		0.92 (0.68–1.25)	1.03 (0.68–1.58)
**When you had sex with this partner, how often did you have sex?**						
Only once or twice ever	Ref	Ref	Ref	Ref	Ref	Ref
Almost every day	0.64 (0.50–0.82) ***	0.68 (0.52–0.87) **	0.63 (0.50–0.80) ***	0.71 (0.54–0.92) **	0.57 (0.45–0.71) ***	0.64 (0.50–0.82) ***
A few times each week	0.69 (0.57–0.83) ***	0.73 (0.60–0.89) **	0.63 (0.52–0.76) ***	0.70 (0.57–0.87) **	0.59 (0.50–0.69) ***	0.66 (0.55–0.79) ***
A few times each month	0.76 (0.65–0.90) **	0.77 (0.65–0.92) **	0.68 (0.57–0.82) ***	0.71 (0.59–0.85) ***	0.64 (0.56–0.75) ***	0.67 (0.57–0.78) ***
Once a month	0.83 (0.71–0.96) *	0.81 (0.69–0.95) **	0.72 (0.61–0.84) ***	0.70 (0.60–0.83) ***	0.70 (0.62–0.80) ***	0.69 (0.60–0.80) ***
Refusal/don’t know	0.66 (0.41–1.01)	0.59 (0.34–1.03)	0.85 (0.63–1.14)	1.04 (0.73–1.48)	0.56 (0.36–0.86) **	0.58 (0.37–0.90) **
**As far as you know, has partner ever had any kind of STI?**						
No	Ref	Ref	Ref	Ref	Ref	Ref
Yes	0.79 (0.65–0.96) **	0.88 (0.72–1.08)	0.73 (0.59–0.90) **	0.95 (0.75–1.20)	0.78 (0.66–0.92)	0.94 (0.79–1.12)
Unknown	0.84 (0.70–1.01) *	0.95 (0.78–1.16)	0.84 (0.70–1.01) *	1.09 (0.87–1.36)	0.86 (0.74–1.00) **	1.03 (0.86–1.23)
**Has partner encouraged you to make sure you use condom when you have sex with other sexual partners?**						
No	Ref	Ref	Ref	Ref	Ref	Ref
Yes	1.15 (1.02–1.31) **	1.09 (0.96–1.24)	1.15 (1.02–1.31) **	1.13 (0.99–1.20)	1.20 (1.07–1.33) **	1.14 (1.02–1.28) **
Don’t know	–	–	–	–	–	–
Refusal	1.28 (1.09–1.51) **	1.00 (0.73–1.36)	1.18 (0.97–1.45)	1.20 (0.91–1.58)	1.31 (1.12–1.53) **	1.04 (0.74–1.45)
**How frequently do you discuss HIV with partner?**						
Never	Ref		Ref	Ref	Ref	Ref
At least once a week	1.25 (1.02–1.53) **	1.17 (0.94–1.45)	1.33 (1.10–1.62) **	1.24 (1.01–1.52) **	1.31 (1.11–1.54) **	1.20 (1.01–1.43) **
Once a month or less	1.26 (1.08–1.47) **	1.24 (1.05–1.46) **	1.03 (0.88–1.22)	0.99 (0.84–1.18)	1.12 (0.98–1.29) *	1.09 (0.95–1.25)
Refused	1.36 (1.15–1.61) ***	1.31 (0.97–1.77)	1.17 (0.97–1.42)	1.05 (0.83–1.35)	1.31 (1.12–1.53) **	1.29 (0.94–1.76)
**As far as you know what your partner’s HIV status is?**						
Negative	Ref		Ref	Ref	Ref	Ref
Positive	0.68 (0.53–0.88) **	0.67 (0.44–1.03)	0.42 (0.31–0.57) ***	0.39 (0.22–0.70) **	0.50 (0.39–0.63) ***	0.43 (0.27–0.68) ***
Unknown	0.77 (0.65–0.92) **	0.79 (0.60–1.04)	0.67 (0.56–0.80) ***	0.75 (0.56–1.00)	0.71 (0.61–0.83) ***	0.74 (0.58–0.94) **
**HIV serostatus concordance**						
Both negative	Ref	Ref	Ref	Ref	Ref	Ref
Both positive	0.68 (0.47–0.97) **	1.07 (0.59–1.95)	0.40 (0.27–0.60) ***	1.22 (0.61–2.45)	0.51 (0.37–0.71) ***	1.39 (0.78–2.49)
HIV serodiscordant/ unknown status	0.87 (0.70–1.07)	0.99 (0.74–1.33)	0.72 (0.57–0.90) **	0.93 (0.69–1.23)	0.80 (0.67–0.96) **	1.02 (0.80–1.30)
Both unknown	0.56 (0.40–0.78) **	0.68 (0.44–1.07)	0.44 (0.32–0.61) ***	0.62 (0.40–0.97) **	0.50 (0.38–0.66) ***	0.71 (0.48–1.04)
**Network size**						
Smaller	Ref	Ref	Ref	Ref	Ref	Ref
Larger	1.41 (1.13–1.76) **	1.47 (1.16–1.84) **	1.40 (1.13–1.75) **	1.51 (1.21–1.90) ***	1.42 (1.17–1.71) **	1.48 (1.22–1.80) ***
Unknown	0.99 (0.71–1.37)	1.19 (0.83–1.71)	1.03 (0.74–1.42)	1.28 (0.90–1.84)	1.15 (0.89–1.50)	1.34 (1.00–1.79)

* *p* ≤ 0.10

** *p* < 0.05

*** *p* < 0.001

In the sub-group analyses for TGW (Supplemental Table 2), results for the multivariable analysis showed that CCU with both types of anal sex was significantly more likely where the participant’s partner had an education at the secondary level or higher, or where the relationship was described as casual, while CCU was significantly less likely with any frequency of sexual acts. In the multivariable analysis among MSM (Supplemental Table 4), CCU was significantly more likely where the relationship was described as casual, where the participant reported that their partner had encouraged the participant to use condoms with their other sexual partners, and a larger network size. Consistent condom use was significantly less likely if the participant’s partner was 45 or older, with any frequency of sexual acts, and if the participants reported that their partners’ HIV status was positive or unknown.

## Discussion

In these analyses among MSM and TGW in Nigeria, the data demonstrated on the individual level, CCU during both insertive and receptive anal sex were positively associated with higher education and disclosure of sexual behavior to a healthcare worker, and on the partnership level was associated with relationship type, partners encouraging condom use, and frequent discussion of HIV by partners. Given that consistent condom use was reported in only 44% of the partnerships reported here and the prevalence of HIV remains high among MSM and TGW in Nigeria, our results highlight possible correlates of condom use which may be leveraged for HIV prevention messaging.

The findings that condom use was more likely in relationships where condom use was encouraged and in relationships where HIV was discussed frequently is consistent with previous studies which highlight that relationship and partner norms influence health behaviors, including condom use [16, 20, 30]. In a study among MSM in the US, it was found that having at least one person in a sexual dyad who did not disapprove of condomless anal sex was associated with an increased likelihood of condomless sex compared to those who did not have such a person in their sexual network [36]. Relationships present opportunities for information exchange and involved parties often influence each other leading to sustained behavior change. This dynamic could be leveraged to create interventions that promote open discussion of safer sex practices. Such interventions may require training for effective communication, as openly discussing sexual behaviors may be uncommon in some communities, particularly ones where HIV and same-sex sexual practices are heavily stigmatized [9, 37].

Results showing that CCU was more likely in casual relationships as compared to regular relationships is also consistent with findings from other studies among MSM where higher rates of unprotected anal intercourse (UAI) have been observed in relationships considered more serious [17, 23, 31]. In these relationships, couples may engage in UAI to increase intimacy and trust in a relationship [15]. While risk perception may decrease with more regular partners, there may be sustained risks for both STIs and HIV in non-exclusive relationships, therefore the importance of prevention modalities should be emphasized for MSM or TGW in non-exclusive relationships. Moreover, previous studies have associated steady partnership as a source of HIV infection among gay men [11, 12]. These findings highlight both the importance and the potential of couple-oriented testing and counseling practices. Couple-based approaches have been shown to be highly effective in several settings and evaluation in the Nigerian context for MSM and TGW may be valuable [14, 38].

Our results also show that participants in relationships where the partners were married to women were less likely to consistently use condoms. The majority of participants in this study reported that they were bisexual and marriage to women was common among their partners. Given the presence of punitive laws prohibiting same sex-relationships in Nigeria, this is unsurprising [37]. Many individuals with gay or bisexual identities may choose to marry women due to fear of stigma, societal pressures, or to avoid being outed [34]. Previous surveys of men who have sex with men and women (MSMW) have shown them to have riskier HIV-related behaviors that potentiate HIV infection, including a higher probability of having an unknown HIV status and a lower probability of encountering HIV prevention activities and materials due to presence of “biphobia” [25]. Thus, MSMW could play a potential role in HIV transmission to women by bridging the epidemics in two different populations. This dynamic could also apply in TGW who have sex with cis-gender women. These results suggest that addressing the prevention needs of MSMW and TGW and all their sex partners is needed for HIV reduction among both KP groups and women. Tailored prevention strategies requires context and understanding of behaviors and sexual networks of TGW who have sex with women and MSMW.

Unlike findings from other studies that have shown age-disparate relationships to be associated with more high-risk HIV-related behaviors including unprotected anal intercourse, our results showed increased odds of condom use during receptive anal sex in relationships where there was a ten year or more difference in age between partners. The risks associated with age-disparate partnerships are often higher in relationships where adolescents have partners who are much older than them. This is due to the increased likelihood for older partners to be living with HIV and a high propensity for condomless sex and alcohol use during sex with older partners [4, 19]. There is also evidence that it may be linked to power dynamics in the relationships [27]. This could lead to a limited ability on the part of younger MSM to adopt or advocate for HIV risk reduction behaviors like condom use even when protected sex is desired. Thus, a ten-year age difference between two middle-aged persons may not be associated with the same risk as a ten-year age difference between an adolescent and a middle-aged person; a nuance which is not sufficiently captured in this analysis and may therefore be the reason for the difference in the results observed.

On the individual level, disclosure of sexual behavior to a healthcare practitioner was also positively associated with CCU. Disclosure of key-population status may indicate more engagement with healthcare services and therefore reduced likelihood for risky behaviors due to more access to targeted HIV prevention messaging. Disclosure of MSM status in Nigeria may be challenged by the presence of laws criminalizing same sex behaviors and the high levels of stigma faced by MSM in the country [9, 37]. Studies have demonstrated that structural elements, where the risks of disclosure of sexual practices for a meaningful risk assessment outweigh the potential benefits of better engagement in HIV prevention services, have affected the uptake of HIV prevention services including condom uptake among MSM at risk for HIV acquisition [9, 37]. This is particularly important as our results showed that participants who reported neither buying condoms nor getting condoms for free were significantly less likely to consistently use condoms, a prevention modality commonly available for free in healthcare centers. Given the importance of the healthcare sector in managing and reducing the HIV epidemic among MSM in the country, training and sensitizing healthcare workers about the impacts of stigmatizing behavior is needed. De-centralization of HIV prevention services by leveraging KP community-engaged facilities such as the trusted community health centers involved in this study may also increase healthcare engagement and alter HIV-related risk behaviors. Longitudinal analysis of data from this cohort showed an improvement in the uptake of condoms and water-based lubricants by MSM and TGW, suggesting effectiveness of these facilities in improving engagement in care [8].

The findings from this study should be interpreted in the context of several limitations. Firstly, all behavioral data collected, including partner and relationship characteristics, were self-reported by the participant in the study. This makes our findings susceptible to multiple biases, including recall and social desirability. The presence of missing data may have biased our results however, missingness was around 1% for many of the variables and is unlikely to significantly impact our interpretation. Additionally, “refusal” and “don’t know” categories were included in models where applicable as these responses may be informative about behavioral risks. Although the study was longitudinal, we carried out cross-sectional analyses using the baseline data. Thus, causality cannot be inferred from our results. While our results may capture the individual and relationship characteristics of MSM and TGW in Lagos and Abuja, Nigeria, our findings may not be generalizable to the broader key population in Nigeria or other countries in sub-Saharan Africa.

## Conclusions

HIV prevention programs may benefit from encouraging open communication of safer sex practices as a strategy for HIV risk reduction given that MSM and TGW were more likely to consistently use condoms when their partners encouraged and openly discussed safer sex. Even with the introduction and scale up of other biomedical HIV prevention modalities such as PrEP, condom use remains an important mode for reducing onward transmission of STIs as HIV and STI infection rates remains sustained among MSM. Implementation science research could support the evaluation of optimal strategies that integrate open discussions about safe sex into existing behavioral interventions. However, while research is helpful, leveraging existing strategies to increase condom use including the provision of condoms in safe spaces, ensuring access to better condoms and condom-compatible lubricants, and teaching condom negotiation skills may support increased consistent condom use among men who have sex with men and transgender women across Nigeria.

## Supplementary Information

**Additional file 1.** Trust Questionnaire.

**Additional file 2: Supplemental Table 1.** Participant characteristics associated with consistent condom use with anal sex among TGW. **Supplemental Table 2.** Partner and relationship characteristics associated with consistent condom use with anal sex among TGW. **Supplemental Table 3.** Participant characteristics associated with consistent condom use with anal sex among MSM. **Supplemental Table 4.** Partner and relationship characteristics associated with consistent condom use with anal sex among MSM.

## Data Availability

In order to protect the clinic staff and the participants from stigma and criminalization of same sex behavior, the data have not been made publicly available. The data are available from the corresponding author on request.

## Abbreviations

AOR: Adjusted odds ratio
CCU: Consistent condom use
CI: Confidence interval
HIV: Human immunodeficiency virus
IQR: Interquartile range
MSM: Men who have sex with men
MSMW: Men who have sex with men and women
N: Number
OR: Odds ratio
P: *p*-value
PrEP: Pre-exposure prophylaxis
RDS: Respondent-driven sampling
SSA: Sub-Saharan Africa
STI: Sexually transmitted infection
TGW: Transgender women
UAI: Unprotected anal intercourse
