# Supplementary material for: Individual and partnership characteristics associated with consistent condom use in a cohort of cisgender men who have sex with men and transgender women in Nigeria
Source: BMC Public Health. 2021 Jun 30;21:1277. doi: 10.1186/s12889-021-11275-w (PMC8243438; doi:10.1186/s12889-021-11275-w)
Supplement: Supplementary file 2 — Additional file 2: Supplemental Table 1. Participant characteristics associated with consistent condom use with anal sex among TGW. Supplemental Table 2. Partner and relationship characteristics associated with consistent condom use with anal sex among TGW. Supplemental Table 3. Participant characteristics associated with consistent condom use with anal sex among MSM. Supplemental Table 4. Partner and relationship characteristics associated with consistent condom use with anal sex among MSM. [file 12889_2021_11275_MOESM2_ESM.docx]

**Supplemental Tables**

Supplemental Table I: Participant characteristics associated with consistent condom use with anal sex among TGW

| Participant characteristics | Associations with insertive anal sex | | Associations with receptive anal sex | | Associations with both types of anal sex | |
| --- | --- | --- | --- | --- | --- | --- |
|  | Bivariate analyses (95% CI) | Multivariable analyses (95% CI) | Bivariate analyses  (95% CI) | Multivariable analyses (95% CI) | Bivariate analyses  (95% CI) | Multivariable analyses (95% CI) |
| **Age categories** |  |  |  |  |  |  |
| 16-19 years | Ref | Ref | Ref | Ref | Ref | Ref |
| 20-24 years | 1.10 (0.30-4.14) | 0.58 (0.16-2.15) | 1.53 (0.79-2.95) | 1.26 (0.62-2.57) | 1.51 (0.81-2.84) | 1.23 (0.63-2.39) |
| 25-29 years | 0.85 (0.21-3.42) | 0.29 (0.06-1.26) | 1.28 (0.61-2.68) | 0.95 (0.42-2.12) | 1.12 (0.55-2.30) | 0.77 (0.35-1.66) |
| 30-34 years | 0.70 (0.12-4.16) | 1.08 (0.11-11.0) | 0.91 (0.29-2.89) | 0.59 (0.18-2.00) | 0.86 (0.29-2.50) | 0.59 (0.20-1.78) |
| 35 and older | 0.80 (0.39-16.67) | 0.12 (0.01-1.48) | 0.78 (0.18-3.36) | 0.42 (0.10-1.76) | 0.84 (0.19-3.85) | 0.37 (0.09-1.54) |
| **Education status** |  |  |  |  |  |  |
| None/primary education only | Ref | Ref | Ref | Ref | Ref | Ref |
| Junior secondary only | 0.22 (0.01-3.17) | 0.09 (0.08-1.06) | 1.86 (0.25-13.4) | 1.44 (0.19-10.8) | 1.69 (0.24-11.8) | 1.29 (0.18-9.40) |
| Senior secondary | 0.54 (0.05-6.38) | 0.16 (0.02-1.65) | 1.64 (0.29-9.29) | 1.04 (0.17-6.31) | 1.67 (0.29-9.44) | 1.04 (0.17-6.34) |
| Higher than secondary | 1.32 (0.10-17.07) | 0.34 (0.35-3.19) | 2.96 (0.50-17.5) | 1.83 (0.30-11.1) | 3.06 (0.52-18.0) | 1.93 (0.32-11.7) |
| Refusal/Don’t know | - | - | - | - | - | - |
| **Sexual orientation** |  |  |  |  |  |  |
| Gay or homosexual | Ref | Ref | Ref | Ref | Ref |  |
| Bisexual | 2.11 (0.95-4.66) | 3.24 (1.08-9.69)** | 1.51 (0.90-2.55) | 1.41 (0.79-2.51) | 1.62 (0.98-2.67) | 1.53 (0.88-2.64) |
| Other | - | - | 0.32 (0.23-0.45)** | 0.12 (0.05-0.28)*** | 0.32 (0.23-0.44)*** | 0.13 (0.06-0.25)*** |
| Refusal/Don’t know | - | - | - | - | - | - |
| **Marital status** |  |  |  |  |  |  |
| Single/Never Married | Ref |  | Ref | Ref | Ref | Ref |
| Married/Cohabitating with a woman | 1.30 (0.31-5.41) |  | 0.65 (0.15-2.72) | 0.55 (0.14-2.24) | 0.65 (0.16-2.60) | 0.56 (0.14-2.25) |
| Cohabitating with a man | 0.20 (0.02-1.71) |  | 0.12 (0.19-0.82)** | 0.14 (0.02-0.87)** | 0.26 (0.07-0.90)** | 0.31 (0.08-1.15) |
| Divorced/Separated/Widowed | - |  | 2.48 (1.57-3.92)*** | 2.75 (1.28-5.94)** | 3.06 (1.82-1.26)*** | 3.60 (1.55-8.36)** |
| **Disclosure to healthcare worker** |  |  |  |  |  |  |
| No | Ref |  | Ref |  | Ref |  |
| Yes | 1.23 (0.53-2.87) |  | 1.01 (0.61-1.68) |  | 1.01 (0.61-1.68) |  |
| Refusal/Don’t know | - |  | - |  | - |  |
| **Heavy Drinking in past 30 days** |  |  |  |  |  |  |
| No | Ref | Ref | Ref |  | Ref |  |
| Yes | 0.33 (0.10-1.16) | 0.32 (0.07-1.42) | 1.04 (0.46-2.36) |  | 0.91 (0.41-2.02) |  |
| Refusal/Don’t know | - |  | 1.05 (0.06-17.2) |  | 0.99 (0.06-16.2) |  |
| **Access to condoms** |  |  |  |  |  |  |
| Gets condoms for free | Ref | Ref | Ref |  | Ref |  |
| Buys condoms | 0.49 (0.19-1.23) | 0.32 (0.11-0.95)** | 1.07 (0.57-2.00) |  | 1.03 (0.57-1.87) |  |
| Gets for free and buys them | 0.86 (0.30-2.43) | 0.48 (0.13-1.75) | 1.61 (0.86-3.04) |  | 1.52 (0.82-2.82) |  |
| Neither | 0.07 (0.01-0.35)** | 0.07 (0.01-0.68)** | 0.48 (0.11-2.12) |  | 0.53 (0.14-1.99) |  |
| Refusal/Don’t know | - | - | - |  | - |  |
| **Knowledge of HIV risks** |  |  |  |  |  |  |
| a.) What type of sex is associated with highest HIV transmission risk? |  |  |  |  |  |  |
| All types of sex carry equal risk | Ref |  | Ref |  | Ref |  |
| Anal sex | 0.51 (0.20-1.28) |  | 0.88 (0.50-1.55) |  | 0.87 (0.50-1.51) |  |
| Vaginal sex | 0.60 0.19-1.92) |  | 0.68 (0.28-1.67) |  | 0.75 (0.33-1.72) |  |
| Oral | - |  | 0.47 (0.04-5.36) |  | 0.46 (0.04-5.23) |  |
| Refusal/Don’t know | 0.35 (0.03-4.20) |  | 0.32 (0.06-1.72) |  | 0.42 (0.10-1.79) |  |
| b.) What type of anal sex is associated with the highest HIV transmission risk? |  |  |  |  |  |  |
| All types of anal sex carry equal risk | Ref |  | Ref |  | Ref |  |
| Receptive anal sex | 0.70 (0.30-1.64) |  | 1.33 (0.77-2.29) |  | 1.35 (0.80-2.27) |  |
| Insertive anal sex | 1.52 (0.36-6.38) |  | 1.33 (0.53-3.32) |  | 1.33 (0.55-3.19) |  |
| Refusal/Don’t know | - |  | - |  | - |  |
| **Any STI test in the past 12 months** |  |  |  |  |  |  |
| No | Ref |  | Ref | Ref | Ref |  |
| Yes | 1.65 (0.72-3.78) |  | 1.45 (0.84-2.50) | 1.19 (0.64-2.21) | 1.37 (0.81-2.30) |  |
| Refusal/Don’t know | 0.99 (0.60-1.62) |  | 0.60 (0.43-0.82)** | 0.47 (0.21-1.06) | 1.15 (0.84-1.56) |  |
| **Any STI diagnosis in the past 12 months** |  |  |  |  |  |  |
| No | Ref |  | Ref |  | Ref |  |
| Yes | 1.27 (0.52-3.11) |  | 1.10 (0.62-1.95) |  | 1.00 (0.58-1.72) |  |
| Refusal/ Don’t know | - |  | - |  | - |  |
| **Worry about HIV in the past 12 months?** |  |  |  |  |  |  |
| Not at all worried | Ref |  | Ref | Ref | Ref |  |
| Somewhat/ a little worried | 1.09 (0.41-2.90) |  | 0.95 (0.51-1.78) |  | 1.03 (0.56-1.89) |  |
| Very worried | 1.23 (0.49-3.11) |  | 0.76 (0.41-1.40) |  | 0.97 (0.54-1.76) |  |
| Refusal/Don’t know | - |  | - |  | - |  |
| **Ever previously tested for HIV?** |  |  |  |  |  |  |
| No | Ref | Ref | Ref | Ref | Ref |  |
| Yes, once | 2.86 (0.86-9.46)* | 1.47 (0.20-10.6) | 1.93 (0.88-4.24) | 2.01 (0.80-5.03) | 1.82 (0.86-3.85) | 1.82 (0.78-4.25) |
| Yes, more than once | 3.71 (1.21-11.4)** | 1.23 (0.21-7.24) | 2.41 (1.17-4.97)** | 2.28 (1.02-5.11)** | 2.27 (1.14-4.53)** | 2.13 (1.03-4.43)** |
| Refusal/Don’t know | - | - | - | - | - | - |
| **Self-reported HIV** |  |  |  |  |  |  |
| Negative | Ref | Ref | Ref |  | Ref |  |
| Positive | 1.28 (0.52-3.16) |  | 1.15 (0.65-2.02) |  | 1.14 (0.66-1.96) |  |
| Unknown | 0.45 (0.16-1.28) |  | 0.55 (0.26-1.14) |  | 0.60 (0.30-1.19) |  |
| **HIV results at baseline** |  |  |  |  |  |  |
| Negative | Ref |  | Ref |  | Ref |  |
| Positive | 0.84 (0.38-1.89) |  | 0.74 (0.43-1.26) |  | 0.71 (0.43-1.18) |  |
| **Site** |  |  |  |  |  |  |
| Abuja | Ref | Ref | Ref |  | Ref |  |
| Lagos | 3.03 (1.33-6.90)** | 4.76 (1.45-15.6)** | 1.29 (0.78-2.12) |  | 1.39 (0.86-2.23) |  |

* p ≤ 0.10

** p < 0.05

*** p < 0.001

Supplemental Table II: Partner and relationship characteristics associated with consistent condom use with anal sex among TGW

| Partner and relationship characteristics | Associations with insertive anal sex | | Associations with receptive anal sex | | Associations with both types of anal sex | |
| --- | --- | --- | --- | --- | --- | --- |
|  | Bivariate analyses  (95% CI) | Multivariable analyses (95% CI) | Bivariate analyses  (95% CI) | Multivariable analyses (95% CI) | Bivariate analyses  (95% CI) | Multivariable analyses (95% CI) |
| **Partner age categories** |  |  |  |  |  |  |
| Younger than 25 | Ref | Ref | Ref | Ref | Ref | Ref |
| 25-34 | 1.21 (0.83-1.74) | 1.40 (0.97-2.03) | 0.96 (0.74-1.24) | 0.85 (0.66-1.11) | 0.91 (0.71-1.17) | 0.82 (0.63-1.07) |
| 35-44 | 0.90 (0.63-1.28) | 1.14 (0.78-1.66) | 0.85 (0.53-1.37) | 0.75 (0.43-1.31) | 0.72 (0.46-1.10) | 0.65 (0.39-1.08) |
| 45 and older | 4.08 (0.04-412.76) | 5.65 (0.13-249.15) | 1.57 (0.38-6.51) | 1.07 (0.21-5.35) | 1.41 (0.36-5.52) | 1.04 (0.21-5.09) |
| Refusal/Don’t know | 0.98 (0.58-1.65) | 1.14 (0.64-2.03) | 1.22 (0.59-2.53) | 0.83 (0.36-1.94) | 1.40 (0.78-2.51) | 0.90 (0.43-1.86) |
| **Partner’s education status** |  |  |  |  |  |  |
| None/Primary education only | Ref | Ref | Ref | Ref | Ref | Ref |
| Junior secondary only | 4.30 (0.52-35.9) | 9.55 (0.81-113.50) | 0.58 (0.17-1.94) | 0.51 (0.15-1.78) | 0.96 (0.36-2.55) | 0.94 (0.35-2.53) |
| Senior secondary | 4.17 (0.54-32.5) | 4.06 (0.35-46.57) | 1.70 (1.00-2.91)* | 1.73 (1.00-2.96)** | 1.74 (1.07-2.83)** | 1.86 (1.09-3.17)** |
| Higher than senior secondary | 3.86 (0.51-29.29) | 3.45 (0.35-34.09) | 2.50 (1.49-4.20)** | 2.66 (1.58-4.47)*** | 2.35 (1.47-3.77)*** | 2.68 (1.61-4.44)*** |
| Refusal/Don’t know | 5.02 (0.60-41.7) | 5.47 (0.53-56.3) | 2.03 (0.93-4.44)* | 1.87 (0.79-4.40) | 2.77 (1.35-5.68)** | 2.76 (1.26-6.07)** |
| **Partner’s sexual orientation** |  |  |  |  |  |  |
| Gay or homosexual | Ref |  | Ref |  | Ref |  |
| Bisexual | 1.16 (0.77-1.74) |  | 1.00 (0.75-1.31) |  | 0.93 (0.72-1.21) |  |
| Refusal/Don’t know | 1.09 (0.71-1.69) |  | 0.63 (0.32-1.25) |  | 0.74 (0.41-1.36) |  |
| **Partner’s marital status** |  |  |  |  |  |  |
| Single/never married | Ref |  | Ref | Ref | Ref | Ref |
| Married to a woman | 1.06 (0.74-1.51) |  | 0.99 (0.71-1.38) | 1.01 (0.68-1.51) | 0.91 (0.66-1.26) | 0.97 (0.66-1.41) |
| Cohabitating with a man | - |  | 0.26 (0.04-1.51) | 0.13 (0.002-6.41) | 0.28 (0.06-1.41) | 0.16 (0.01-4.72) |
| Separated/divorced/widowed | - |  | 0.91 (0.08-10.2) | 0.64 (0.06-6.42) | 0.90 (0.08-10.0) | 0.60 (0.05-7.92) |
| Refusal/Don’t know | - |  | 2.60 (1.15-5.86)** | 3.08 (1.04-9.17)** | 2.53 (1.11-5.74)** | 3.31 (1.12-9.75)** |
| **Relationship type** |  |  |  |  |  |  |
| Regular | Ref |  | Ref | Ref | Ref | Ref |
| Casual | 1.26 (0.90-1.79) |  | 1.70 (1.31-2.21)*** | 1.65 (1.20-2.25)** | 1.66 (1.30-2.11)*** | 1.55 (1.15-2.09)** |
| Refusal/Don’t know | - |  | - |  | - | - |
| **Age Difference between partner and participant** |  |  |  |  |  |  |
| No age difference | Ref |  | Ref | Ref | Ref | Ref |
| Greater than or equal to ten years | 0.76 (0.47-1.24) |  | 0.78 (0.51-1.18) | 0.74 (0.41-1.33) | 0.72 (0.50-1.06)* | 0.73 (0.42-1.26) |
| Less than ten years | 0.85 (0.59-1.22) |  | 0.75 (0.55-1.02)* | 0.70 (0.47-1.06) | 0.73 (0.54-0.98)** | 0.68 (0.48-0.98) |
| Unknown age difference | 0.74 (0.40-1.38) |  | 0.97 (0.44-2.12) | - | 1.12 (0.58-2.17) | - |
| **Partner Age mix** |  |  |  |  |  |  |
| All partner’s ages were different | Ref |  | Ref |  | Ref |  |
| All partners were consistently younger than participant | 0.52 (0.13-2.06) |  | 0.96 (0.32-2.89) |  | 0.81 (0.30-2.18) |  |
| All partners were consistently older than participant | 0.63 (0.26-1.52) |  | 0.76 (0.44-1.30) |  | 0.70 (0.42-1.17) |  |
| All partners were the same age as participant | - |  |  |  | - |  |
| Unknown age mix | 0.65 (0.08-5.01) |  | 1.20 (0.25-5.76) |  | 1.11 (0.23-5.31) |  |
| **Partner socio-economic status** |  |  |  |  |  |  |
| Same as participant’s | Ref |  | Ref |  | Ref |  |
| Lower than participant’s | 1.02 (0.56-1.86) |  | 0.78 (0.51-1.19) |  | 0.88 (0.59-1.30) |  |
| Higher than participant’s | 1.06 (0.61-1.86) |  | 0.95 (0.61-1.48) |  | 0.97 (0.65-1.46) |  |
| Refusal/don’t know | - |  | 1.15 (0.70-1.89) |  | 1.21 (0.73-2.00) |  |
| **Did your partner have more than one regular sexual partner at the same time?** |  |  |  |  |  |  |
| No | Ref |  | Ref |  | Ref |  |
| Yes | 0.93 (0.58-1.51) |  | 1.24 (0.88-1.76) |  | 1.14 (0.82-1.58) |  |
| Don’t know | 1.70 (0.87-3.34) |  | 1.29 (0.70-2.38) |  | 1.17 (0.67-2.05) |  |
| **How many sexual partners do you believe your partner has had in the past year?** |  |  |  |  |  |  |
| No other sexual partners | Ref |  | Ref |  | Ref |  |
| 1-2 sexual partners | 1.32 (0.55-3.18) |  | 1.10 (0.52-2.36) |  | 1.04 (0.49-2.23) |  |
| 3-5 sexual partners | 1.68 (0.73-3.90) |  | 1.43 (0.67-3.02) |  | 1.47 (0.69-3.13) |  |
| Greater than 5 | 2.52 (0.56-11.3) |  | 1.24 (0.52-2.94) |  | 1.29 (0.56-3.00) |  |
| Don’t know | 1.31 (0.54-3.19) |  | 1.33 (0.63-2.79) |  | 1.37 (0.65-2.88) |  |
| **Do you believe partner has ever had sex under the influence of drug or alcohol?** |  |  |  |  |  |  |
| No | Ref |  | Ref |  | Ref |  |
| Yes | 1.10 (0.58-2.09) |  | 0.89 (0.57-1.38) |  | 0.91 (0.61-1.38) |  |
| Don’t know | 1.17 (0.47-2.90) |  | 0.64 (0.27-1.55) |  | 0.82 (0.39-1.71) |  |
| Refusal | 1.78 (0.58-5.53) |  | 1.12 (0.58-2.14) |  | 1.31 (0.70-2.44) |  |
| **When partner has sex with other sexual partners how often do you believe a condom is used** |  |  |  |  |  |  |
| Never | Ref | Ref | Ref |  | Ref |  |
| Sometimes | 0.54 (0.25-1.17) | 0.43 (0.23-0.82)** | 0.96 (0.47-1.92) |  | 1.04 (0.54-2.00) |  |
| Almost always | 0.90 (0.15-5.59) | 0.48 (0.06-4.02) | 0.64 (0.24-1.76) |  | 0.75 (0.28-2.02) |  |
| Always | 0.36 (0.16-0.84)** | 0.24 (0.08-0.66)** | 1.13 (0.56-2.27) |  | 1.09 (0.56-2.11) |  |
| Don’t know | 0.50 (0.21-1.21) | 0.37 (0.13-1.02) | 1.07 (0.50-2.30) |  | 1.08 (0.53-2.20) |  |
| Refusal | 0.76 (0.38-1.54) | 0.53 (0.19-1.46) | 1.12 (0.53-2.40) |  | 1.29 (0.65-2.57) |  |
| **When you had sex with this partner, how often did you have sex?** |  |  |  |  |  |  |
| Only once or twice ever | Ref | Ref | Ref | Ref | Ref | Ref |
| Almost every day | 0.57 (0.31-1.08)* | 0.83 (0.48-1.45) | 0.32 (0.14-0.73)** | 0.51 (0.23-1.10) | 0.35 (0.17-0.72)** | 0.54 (0.26-1.11) |
| A few times each week | 0.56 (0.21-1.47) | 0.70 (0.30-1.64) | 0.31 (0.165-0.59)*** | 0.42 (0.23-0.78)** | 0.34 (0.18-0.63)** | 0.46 (0.25-0.86)** |
| A few times each month | 0.63 (0.30-1.31) | 0.77 (0.39-1.53) | 0.42 (0.24-0.77)** | 0.50 (0.28-0.90)** | 0.42 (0.24-0.74)** | 0.50 (0.29-0.88)** |
| Once a month | 0.90 (0.47-1.72) | 1.10 (0.63-1.93) | 0.39 (0.22-0.71) | 0.38 (0.21-0.69)** | 0.44 (0.25-0.78)** | 0.44 (0.25-0.78)** |
| Refusal/don’t know | 0.70 (0.17-2.81) | 0.85 (0.36-2.05) | - | - | 0.62 (0.28-1.39) | 0.48 (0.27-0.84)** |
| **As far as you know, has partner ever had any kind of STI?** |  |  |  |  |  |  |
| No | Ref |  | Ref |  | Ref |  |
| Yes | 0.87 (0.41-1.84) |  | 0.80 (0.50-1.29) |  | 0.78 (0.49-1.24) |  |
| Unknown | 1.34 (0.55-3.23) |  | 1.17 (0.75-1.83) |  | 1.10 (0.69-1.76) |  |
| **Has partner encouraged you to make sure you use condom when you have sex with other sexual partners?** |  |  |  |  |  |  |
| No | Ref |  | Ref |  | Ref |  |
| Yes | 0.76 (0.47-1.24) |  | 0.92 (0.67-1.26) |  | 0.89 (0.67-1.19) |  |
| Don’t know | - |  | - |  | - |  |
| Refusal | 0.94 (0.86-2.22) |  | 1.03 (0.66-1.61) |  | 1.07 (0.70-1.64) |  |
| **How frequently do you discuss HIV with partner?** |  |  |  |  |  |  |
| Never | Ref |  | Ref |  | Ref |  |
| At least once a week | 1.98 (1.01-3.86) ** | 2.25 (1.03-4.91)** | 1.22 (0.75-1.99) |  | 1.40 (0.88-2.22) |  |
| Once a month or less | 1.24 (0.59-2.59) | 1.49 (0.72-3.05) | 1.13 (0.76-1.67) |  | 1.22 (0.84-1.78) |  |
| Refused | 1.38 (0.80-2.37) | 1.35 (0.65-2.83) | 1.17 (0.71-1.91) |  | 1.33 (0.82-2.14) |  |
| **As far as you know what your partner’s HIV status is?** |  |  |  |  |  |  |
| Negative | Ref |  | Ref |  | Ref |  |
| Positive | 1.20 (0.26-5.54) |  | 0.71 (0.30-1.67) |  | 0.68 (0.29-1.60) |  |
| Unknown | 1.14 (0.58-2.23) |  | 0.92 (0.54-1.58) |  | 0.89 (0.52-1.50) |  |
| **HIV serostatus concordance** |  |  |  |  |  |  |
| Both negative | Ref |  | Ref |  | Ref |  |
| Both positive | 1.97 (0.41-9.56) |  | 0.92 (0.32-2.66) |  | 1.08 (0.37-3.10) |  |
| HIV serodiscordant/ unknown status | 0.93 (0.33-2.64) |  | 1.21 (0.69-2.10) |  | 1.14 (0.62-2.08) |  |
| Both unknown | 1.04 (0.18-5.99) |  | 0.57 (0.20-1.61) |  | 0.53 (0.19-1.45) |  |
| **Network size** |  |  |  |  |  |  |
| Smaller | Ref |  | Ref |  | Ref |  |
| Larger | 1.31 (0.57-3.01) |  | 0.95 (0.53-1.72) |  | 0.98 (0.56-1.73) |  |
| Unknown | 0.58 (0.09-3.78) |  | 0.94 (0.37-2.37) |  | 1.03 (0.43-2.47) |  |

* p ≤ 0.10

** p < 0.05

*** p < 0.001

Supplemental Table III: Participant characteristics associated with consistent condom use with anal sex among MSM

| Participant characteristics | Associations with insertive anal sex | | Associations with receptive anal sex | | Associations with both types of anal sex | |
| --- | --- | --- | --- | --- | --- | --- |
|  | Bivariate analyses (95% CI) | Multivariable analyses (95% CI) | Bivariate analyses  (95% CI) | Multivariable analyses (95% CI) | Bivariate analyses  (95% CI) | Multivariable analyses (95% CI) |
| **Age categories** |  |  |  |  |  |  |
| 16-19 years | Ref | Ref | Ref | Ref | Ref | Ref |
| 20-24 years | 0.69 (0.47-1.00)* | 0.54 (0.36-0.81)** | 0.94 (0.68-1.29) | 0.71 (0.50-1.01) | 0.88 (0.66-1.17) | 0.70 (0.51-0.96)** |
| 25-29 years | 0.57 (0.39-0.84)** | 0.39 (0.25-0.60)*** | 0.81 (0.57-1.14) | 0.52 (0.36-0.77)** | 0.74 (0.54-1.01)* | 0.53 (0.38-0.75)*** |
| 30-34 years | 0.71 (0.45-1.15) | 0.52 (0.31-0.88)** | 0.99 (0.62-1.60) | 0.68 (0.40-1.14) | 0.89 (0.60-1.32) | 0.67 (0.44-1.04) |
| 35 and older | 0.66 (0.38-1.15) | 0.54 (0.29-1.00)* | 0.74 (0.38-1.44) | 0.54 (0.25-1.14) | 0.87 (0.53-1.42) | 0.71 (0.42-1.22) |
| **Education status** |  |  |  |  |  |  |
| None/primary education only | Ref | Ref | Ref | Ref | Ref | Ref |
| Junior secondary only | 1.20 (0.56-2.59) | 1.14 (0.49-2.64) | 1.18 (0.58-2.42) | 0.96 (0.44-2.09) | 1.49 (0.79-2.81) | 1.42 (0.71-2.83) |
| Senior secondary | 2.76 (1.58-4.85)*** | 2.29 (1.21-4.34)** | 1.68 (0.97-2.93)* | 1.23 (0.66-2.30) | 2.19 (1.35-3.57)** | 1.77 (1.03-3.05)** |
| Higher than secondary | 2.79 (1.58-4.90)*** | 2.55 (1.34-4.88)** | 2.09 (1.19-3.68)** | 1.65 (0.88-3.10) | 2.37 (1.45-3.89)** | 2.07 (1.20-3.59)** |
| Refusal/Don’t know | 1.42 (0.24-8.46) | 1.24 (0.20-7.77) | 1.41 (0.21-9.29) | 1.01 (0.14-7.34) | 0.99 (0.18-5.52) | 0.82 (0.15-4.40) |
| **Sexual orientation** |  |  |  |  |  |  |
| Gay or homosexual | Ref |  | Ref |  | Ref |  |
| Bisexual | 1.17 (0.90-1.51) |  | 1.00 (0.79-1.28) |  | 1.06 (0.86-1.31) |  |
| Other | - |  | - |  | - |  |
| Refusal/Don’t know | 4.04 (0.64-25.6) |  | 1.32 (0.08-21.3) |  | 2.89 (0.41-20.5) |  |
| **Marital status** |  |  |  |  |  |  |
| Single/Never Married | Ref |  | Ref |  | Ref |  |
| Married/Cohabitating with a woman | 0.94 (0.60-1.50) |  | 1.00 (0.60-1.67) |  | 0.98 (0.65-1.50) |  |
| Cohabitating with a man | 1.79 (0.74-4.30) |  | 1.31 (0.63-2.74) |  | 1.25 (0.64-2.44) |  |
| Divorced/Separated/Widowed | 0.57 (0.28-1.18) |  | 0.94 (0.49-1.82) |  | 0.77 (0.42-1.40) |  |
| **Disclosure to healthcare worker** |  |  |  |  |  |  |
| No | Ref | Ref | Ref | Ref | Ref |  |
| Yes | 1.72 (1.36-2.18)*** | 1.41 (1.09-1.82)** | 1.73 (1.36-2.18)*** | 1.46 (1.12-1.90)** | 1.56 (1.28-1.90)*** |  |
| Refusal/Don’t know | 0.84 (0.15-4.78) | 1.11 (0.15-8.13) | 0.21 (0.04-1.03)* | 0.12 (0.04-0.38) | 0.62 (0.10-3.99) |  |
| **Heavy Drinking in past 30 days** |  |  |  |  |  |  |
| No | Ref |  | Ref |  | Ref |  |
| Yes | 1.36 (0.93-1.98) |  | 1.22 (0.77-1.92) |  | 1.23 (0.87-1.74) |  |
| Refusal/Don’t know | 0.83 (0.41-1.69) |  | 0.93 (0.39-2.25) |  | 0.81 (0.41-1.58) |  |
| **Access to condoms** |  |  |  |  |  |  |
| Gets condoms for free | Ref | Ref | Ref | Ref | Ref | Ref |
| Buys condoms | 0.71 (0.55-0.93)** | 0.88 (0.66-1.16) | 0.68 (0.52-0.89)** | 0.86 (0.64-1.14) | 0.75 (0.59-0.94)** | 0.91 (0.72-1.16) |
| Gets for free and buys them | 0.87 (0.65-1.16) | 0.81 (0.61-1.10) | 0.77 (0.57-1.02)* | 0.76 (0.56-1.03) | 0.81 (0.63-1.03)* | 0.78 (0.60-1.00) |
| Neither | 0.16 (0.05-0.55)** | 0.29 (0.07-1.10) | 0.08 (0.02-0.33)** | 0.11 (0.02-0.50)** | 0.14 (0.05-0.41)*** | 0.21 (0.07-0.65)** |
| Refusal/Don’t know | 0.82 (0.22-3.09) | 1.78 (0.31-10.1) | 0.96 (0.30-3.05) | 1.54 (0.40-5.92) | 0.80 (0.27-2.33) | 1.40 (0.41-4.78) |
| **Knowledge of HIV risks** |  |  |  |  |  |  |
| a.) What type of sex is associated with highest HIV transmission risk? |  |  |  |  |  |  |
| All types of sex carry equal risk | Ref | Ref | Ref | Ref | Ref | Ref |
| Anal sex | 1.46 (1.10-1.92)** | 1.31 (0.98-1.75) | 1.50 (1.15-1.95)** | 1.31 (0.99-1.74) | 1.38 (1.10-1.74)** | 1.24 (0.98-1.58) |
| Vaginal sex | 1.22 (0.88-1.71) | 1.30 (0.90-1.87) | 1.38 (0.94-2.04) | 1.66 (1.10-2.50)** | 1.16 (0.86-1.56) | 1.26 (0.92-1.73) |
| Oral | 1.26 (0.70-2.27) | 1.65 (0.85-3.20) | 2.01 (1.15-3.50)** | 2.17 (1.20-3.93)** | 1.68 (1.03-2.73)** | 1.92 (1.16-3.19)** |
| Refusal/Don’t know | 0.70 (0.35-1.38) | 1.17 (0.58-2.36) | 0.97 (0.49-1.91) | 1.53 (0.40-5.92) | 0.73 (0.40-1.34) | 0.98 (0.53-1.82) |
| b.) What type of anal sex is associated with the highest HIV transmission risk? |  |  |  |  |  |  |
| All types of anal sex carry equal risk | Ref |  | Ref |  | Ref |  |
| Receptive anal sex | 1.01 (0.79-1.28) |  | 1.08 (0.85-1.36) |  | 1.02 (0.84-1.25) |  |
| Insertive anal sex | 1.09 (0.74-1.61) |  | 1.19 (0.76-1.87) |  | 1.17 (0.84-1.64) |  |
| Refusal/Don’t know | 0.77 (0.45-1.33) |  | 0.83 (0.44-1.54) |  | 0.86 (0.53-1.39) |  |
| **Any STI test in the past 12 months** |  |  |  |  |  |  |
| No | Ref | Ref | Ref | Ref | Ref | Ref |
| Yes | 1.31 (1.03-1.64)** | 1.15 (0.90-1.46) | 1.32 (1.05-1.68)** | 1.15 (0.89-1.48) | 1.25 (1.03-1.52)** | 1.10 (0.90-1.36) |
| Refusal/Don’t know | - | - | - |  | - | - |
| **Any STI diagnosis in the past 12 months** |  |  |  |  |  |  |
| No | Ref |  | Ref |  | Ref |  |
| Yes | 0.90 (0.69-1.16) |  | 0.82 (0.62-1.07) |  | 0.85 (0.69-1.06) |  |
| Refusal/ Don’t know | 0.52 (0.13-2.08) |  | 0.42 (0.08-2.28) |  | 0.52 (0.15-1.79) |  |
| **Worry about HIV in the past 12 months?** |  |  |  |  |  |  |
| Not at all worried | Ref | Ref | Ref | Ref | Ref | Ref |
| Somewhat/ a little worried | 0.96 (0.74-1.26) | 0.86 (0.65-1.14) | 1.07 (0.82-1.40) | 0.99 (0.74-1.31) | 0.95 (0.76-1.20) | 0.86 (0.68-1.09) |
| Very worried | 0.74 (0.57-0.97)** | 0.64 (0.48-0.85)** | 0.78 (0.59-1.03)* | 0.69 (0.51-0.92)** | 0.75 (0.59-0.94)** | 0.65 (0.51-0.83)*** |
| Refusal/Don’t know | 0.55 (0.47-0.65)*** | 0.71 (0.46-1.09) | - |  | 1.89 (0.27-13.2) | 2.41 (0.33-17.8) |
| **Ever previously tested for HIV?** |  |  |  |  |  |  |
| No | Ref | Ref | Ref | Ref | Ref | Ref |
| Yes, once | 1.50 (1.06-2.12)** | 0.92 (0.45-1.92) | 1.59 (1.13-2.21)** | 1.78 (0.88-3.60) | 1.56 (1.16-2.09)** | 1.32 (0.73-2.36) |
| Yes, more than once | 1.97 (1.46-2.67)*** | 1.17 (0.56-2.48) | 1.82 (1.36-2.45)*** | 1.95 (0.93-4.09) | 1.80 (1.40-2.32)*** | 1.45 (0.79-2.67) |
| Refusal/Don’t know | 13.8 (2.77-68.8)* | 27.4 (10.1-74.6)*** | 5.66 (4.39-7.28)*** | 9.53 (4.45-20.4)*** | 13.36 (2.70-66.1)** | 22.2 (7.69-64.1)*** |
| **Self-reported HIV** |  |  |  |  |  |  |
| Negative | Ref | Ref | Ref | Ref | Ref | Ref |
| Positive | 1.34 (1.03-1.74)** | 1.21 (0.91-1.61) | 1.30 (1.00-1.68)** | 1.22 (0.91-1.62) | 1.25 (1.00-1.54)** | 1.14 (0.90-1.45) |
| Unknown | 0.61 (0.46-0.81)** | 0.73 (0.36-1.46) | 0.71 (0.53-0.94)** | 1.43 (0.72-2.84) | 0.66 (0.52-0.84)** | 0.97 (0.55-1.70) |
| **HIV results at baseline** |  |  |  |  |  |  |
| Negative | Ref |  | Ref |  | Ref |  |
| Positive | 0.89 (0.71-1.11) |  | 0.86 (0.68-1.08) |  | 0.90 (0.75-1.10) |  |
| **Site** |  |  |  |  |  |  |
| Abuja | Ref | Ref | Ref | Ref | Ref | Ref |
| Lagos | 1.91 (1.49-2.44)*** | 1.59 (1.21-2.09)** | 1.45 (1.16-1.82)** | 1.21 (0.93-1.59) | 1.58 (1.30-1.92)*** | 1.38 (1.10-1.72)** |

Supplemental Table IV: Partner and relationship characteristics associated with consistent condom use with anal sex among MSM

| Partner and relationship characteristics | Associations with insertive anal sex | | Associations with receptive anal sex | | Associations with both types of anal sex | |
| --- | --- | --- | --- | --- | --- | --- |
|  | Bivariate analyses  (95% CI) | Multivariable analyses (95% CI) | Bivariate analyses  (95% CI) | Multivariable analyses (95% CI) | Bivariate analyses  (95% CI) | Multivariable analyses (95% CI) |
| **Partner age categories** |  |  |  |  |  |  |
| Younger than 25 | Ref | Ref | Ref | Ref | Ref | Ref |
| 25-34 | 0.92 (0.83-1.02) | 0.88 (0.79-0.98)** | 1.03 (0.91-1.17) | 0.95 (0.83-1.09) | 1.01 (0.91-1.12) | 0.97 (0.87-1.08) |
| 35-44 | 0.90 (0.75-1.08) | 0.87 (0.72-1.05) | 1.00 (0.81-1.23) | 0.87 (0.70-1.09) | 0.95 (0.79-1.13) | 0.91 (0.75-1.09) |
| 45 and older | 0.86 (0.66-1.13) | 0.79 (0.60-1.05) | 0.70 (0.50-0.98)** | 0.59 (0.41-0.86) | 0.77 (0.56-1.04)* | 0.72 (0.51-1.00)** |
| Refusal/Don’t know | 0.90 (0.72-1.14) | 0.96 (0.74-1.26) | 0.82 (0.64-1.05) | 0.88 (0.67-1.16) | 0.86 (0.70-1.05) | 0.94 (0.75-1.19) |
| **Partner’s education status** |  |  |  |  |  |  |
| None/Primary education only | Ref | Ref | Ref | Ref | Ref | Ref |
| Junior secondary only | 0.85 (0.62-1.15) | 0.87 (0.63-1.20) | 1.03 (0.71-1.50) | 0.99 (0.67-1.46) | 0.93 (0.66-1.31) | 0.95 (0.67-1.34) |
| Senior secondary | 1.07 (0.81-1.42) | 1.11 (0.82-1.49) | 1.14 (0.85-1.54) | 1.16 (0.85-1.58) | 1.12 (0.86-1.47) | 1.17 (0.88-1.54) |
| Higher than senior secondary | 1.04 (0.79-1.38) | 1.15 (0.85-1.54) | 1.30 (0.95-1.78)* | 1.36 (0.96-1.86) | 1.18 (0.89-1.54) | 1.26 (0.95-1.68) |
| Refusal/Don’t know | 1.10 (0.77-1.57) | 1.23 (0.85-1.79) | 1.11 (0.77-1.61) | 1.23 (0.83-1.81) | 1.09 (0.79-1.50) | 1.20 (0.86-1.68) |
| **Partner’s sexual orientation** |  |  |  |  |  |  |
| Gay or homosexual | Ref |  | Ref |  | Ref |  |
| Bisexual | 0.98 (0.88-1.08) |  | 1.05 (0.92-1.20) |  | 0.98 (0.88-1.09) |  |
| Refusal/Don’t know | 1.01 (0.80-1.28) |  | 1.10 (0.82-1.46) |  | 0.96 (0.76-1.22) |  |
| **Partner’s marital status** |  |  |  |  |  |  |
| Single/never married | Ref |  | Ref |  | Ref |  |
| Married to a woman | 0.99 (0.88-1.11) |  | 0.96 (0.82-1.12) |  | 0.96 (0.85-1.09) |  |
| Cohabitating with a man | 0.55 (0.10-3.12) |  | 0.34 (0.07-1.72) |  | 0.33 (0.07-1.55) |  |
| Separated/divorced/widowed | 1.06 (0.73-1.54) |  | 0.93 (0.59-1.47) |  | 1.07 (0.67-1.70) |  |
| Refusal/Don’t know | 1.29 (0.88-1.88) |  | 0.98 (0.59-1.62) |  | 1.33 (0.89-1.98) |  |
| **Relationship type** |  |  |  |  |  |  |
| Regular | Ref | Ref | Ref | Ref | Ref | Ref |
| Casual | 1.24 (1.13-1.36)*** | 1.14 (1.03-1.27)** | 1.25 (1.13-1.39)*** | 1.19 (1.05-1.36)** | 1.28 (1.17-1.40)*** | 1.18 (1.06-1.31)** |
| Refusal/Don’t know | 0.56 (0.08-4.03) | 0.49 (0.05-4.54) | 1.27 (0.73-2.21) | 0.79 (0.56-1.10) | 0.73 (0.11-4.95) | 0.73 (0.07-7.28) |
| **Age Difference between partner and participant** |  |  |  |  |  |  |
| No age difference | Ref |  | Ref |  | Ref |  |
| Greater than or equal to ten years | 0.98 (0.84-1.15) |  | 1.13 (0.92-1.40) |  | 1.05 (0.89-1.23) |  |
| Less than ten years | 0.94 (0.84-1.06) |  | 1.07 (0.91-1.26) |  | 1.01 (0.89-1.14) |  |
| Unknown age difference | 0.91 (0.70-1.17) |  | 0.88 (0.67-1.16) |  | 0.88 (0.70-1.10) |  |
| **Partner Age mix** |  |  |  |  |  |  |
| All partner’s ages were different | Ref | Ref | Ref | Ref | Ref | Ref |
| All partners were consistently younger than participant | 1.03 (0.77-1.39) | 1.10 (0.81-1.50) | 0.91 (0.61-1.35) | 0.95 (0.63-1.43) | 1.08 (0.82-1.43) | 1.13 (0.85-1.49) |
| All partners were consistently older than participant | 1.08 (0.83-1.41) | 1.16 (0.88-1.52) | 1.06 (0.83-1.35) | 1.03 (0.80-1.33) | 1.06 (0.86-1.31) | 1.08 (0.87-1.34) |
| All partners were the same age as participant | 0.74 (0.26-2.12) | 0.76 (0.27-2.19) | 2.35 (0.79-7.01) | 2.62 (0.92-7.47) | 1.14 (0.49-2.65) | 1.14 (0.50-2.64) |
| Unknown age mix | 0.49 (0.28-0.84)** | 0.61 (0.32-1.13) | 0.42 (0.23-0.74)** | 0.52 (0.28-1.00)** | 0.50 (0.31-0.80)** | 0.65 (0.38-1.11) |
| **Partner socio-economic status** |  |  |  |  |  |  |
| Same as participant’s | Ref |  | Ref | Ref | Ref |  |
| Lower than participant’s | 1.04 (0.89-1.20) |  | 1.03 (0.85-1.25) | 1.03 (0.83-1.26) | 1.05 (0.91-1.22) |  |
| Higher than participant’s | 0.93 (0.82-1.07) |  | 1.12 (0.95-1.32) | 1.14 (0.96-1.35) | 1.01 (0.89-1.15) |  |
| Refusal/don’t know | 1.01 (0.68-1.48) |  | 0.67 (0.44-1.01)* | 0.88 (0.58-1.35) | 0.90 (0.64-1.28) |  |
| **Did your partner have more than one regular sexual partner at the same time?** |  |  |  |  |  |  |
| No | Ref |  | Ref |  | Ref |  |
| Yes | 1.17 (0.97-1.41) |  | 1.04 (0.86-1.26) |  | 1.09 (0.93-1.27) |  |
| Don’t know | 1.06 (0.79-1.43) |  | 1.03 (0.76-1.41) |  | 1.02 (0.79-1.31) |  |
| **How many sexual partners do you believe your partner has had in the past year?** |  |  |  |  |  |  |
| No other sexual partners | Ref | Ref | Ref | Ref | Ref | Ref |
| 1-2 sexual partners | 1.14 (0.70-1.84) | 1.16 (0.73-1.87) | 1.51 (0.94-2.44)* | 1.51 (0.94-2.44) | 1.32 (0.83-2.09) | 1.29 (0.80-2.08) |
| 3-5 sexual partners | 1.40 (0.90-2.19) | 1.47 (0.94-2.31) | 1.89 (1.21-2.94)** | 1.95 (1.24-3.06)** | 1.55 (1.00-2.42)* | 1.58 (1.00-2.49) |
| Greater than 5 | 1.63 (1.02-2.62)** | 1.72 (1.06-2.77)** | 1.55 (0.97-2.48)* | 1.64 (1.02-2.67)** | 1.51 (0.95-2.40)* | 1.58 (0.98-2.56) |
| Don’t know | 1.32 (0.86-2.04) | 1.59 (1.03-2.46)** | 1.40 (0.89-2.19) | 1.67 (1.05-2.64)** | 1.32 (0.85-2.04) | 1.52 (0.96-2.41) |
| **Do you believe partner has ever had sex under the influence of drug or alcohol?** |  |  |  |  |  |  |
| No | Ref | Ref | Ref | Ref | Ref | Ref |
| Yes | 1.14 (0.95-1.37) | 1.09 (0.90-1.33) | 0.95 (0.79-1.13) | 0.96 (0.79-1.16) | 1.00 (0.86-1.17) | 1.00 (0.84-1.17) |
| Don’t know | 0.89 (0.65-1.22) | 0.85 (0.62-1.17) | 0.83 (0.62-1.11) | 0.89 (0.65-1.23) | 0.84 (0.64-1.10) | 0.85 (0.64-1.13) |
| Refusal | 0.63 (0.49-0.81)*** | 0.59 (0.44-0.79)*** | 0.62 (0.48-0.80)*** | 0.64 (0.46-0.88)** | 0.66 (0.54-0.82)*** | 0.62 (0.39-0.97)** |
| **When partner has sex with other sexual partners how often do you believe a condom is used** |  |  |  |  |  |  |
| Never | Ref |  | Ref |  | Ref | Ref |
| Sometimes | 0.79 (0.49-1.29) |  | 1.04 (0.68-1.59) |  | 1.01 (0.71-1.43) | 0.87 (0.61-1.25) |
| Almost always | 0.89 (0.52-1.54) |  | 0.82 (0.50-1.34) |  | 0.89 (0.60-1.34) | 0.79 (0.52-1.20) |
| Always | 1.13 (0.71-1.81) |  | 1.24 (0.82-1.88) |  | 1.34 (0.95-1.89)* | 1.14 (0.79-1.64) |
| Don’t know | 0.92 (0.56-1.50) |  | 0.91 (0.57-1.46) |  | 1.05 (0.72-1.53) | 1.08 (0.73-1.61) |
| Refusal | 0.81 (0.51-1.29) |  | 0.82 (0.52-1.31) |  | 0.95 (0.66-1.37) | 1.08 (0.64-1.80) |
| **When you had sex with this partner, how often did you have sex?** |  |  |  |  |  |  |
| Only once or twice ever | Ref | Ref | Ref | Ref | Ref | Ref |
| Almost every day | 0.63 (0.49-0.82)** | 0.69 (0.53-0.91)** | 0.62 (0.48-0.78)*** | 0.68 (0.51-0.91)** | 0.53 (0.42-0.68)*** | 0.58 (0.45-0.76)*** |
| A few times each week | 0.69 (0.57-0.84)*** | 0.77 (0.61-0.95)** | 0.66 (0.54-0.82)*** | 0.74 (0.58-0.95)** | 0.60 (0.50-0.72)*** | 0.67 (0.54-0.82)*** |
| A few times each month | 0.77 (0.64-0.92)** | 0.79 (0.65-0.96)** | 0.69 (0.57-0.85)*** | 0.72 (0.58-0.90)** | 0.65 (0.55-0.77)*** | 0.67 (0.56-0.81)*** |
| Once a month | 0.82 (0.69-0.97)** | 0.83 (0.69-0.99)** | 0.73 (0.61-0.88)** | 0.74 (0.61-0.90)** | 0.71 (0.61-0.83)*** | 0.72 (0.61-0.84)*** |
| Refusal/don’t know | 0.52 (0.27-1.00)** | 0.48 (0.23-0.97)** | - | - | 0.40 (0.19-0.83)** | 0.37 (0.18-0.78)** |
| **As far as you know, has partner ever had any kind of STI?** |  |  |  |  |  |  |
| No | Ref | Ref | Ref | Ref | Ref | Ref |
| Yes | 0.80 (0.66-0.98)** | 0.89 (0.72-1.11) | 0.69 (0.54-0.88)** | 0.92 (0.70-1.20) | 0.76 (0.63-0.91)** | 0.90 (0.74-1.10) |
| Unknown | 0.80 (0.66-0.97)** | 0.89 (0.72-1.11) | 0.77 (0.62-0.95)** | 1.02 (0.78-1.33) | 0.80 (0.68-0.95)** | 0.95 (0.78-1.16) |
| **Has partner encouraged you to make sure you use condom when you have sex with other sexual partners?** |  |  |  |  |  |  |
| No | Ref | Ref | Ref | Ref | Ref | Ref |
| Yes | 1.19 (1.03-1.37)** | 1.13 (0.98-1.32) | 1.23 (1.05-1.43)** | 1.23 (1.05-1.45)** | 1.27 (1.11-1.44)*** | 1.22 (1.07-1.38)** |
| Don’t know | - | - | - | - | - | - |
| Refusal | 1.32 (1.10-1.58)** | 1.08 (0.78-1.49) | 1.30 (1.00-1.68)** | 1.29 (0.94-1.77) | 1.37 (1.14-1.65)** | 1.02 (0.71-1.47) |
| **How frequently do you discuss HIV with partner?** |  |  |  |  |  |  |
| Never | Ref | Ref | Ref | Ref | Ref | Ref |
| At least once a week | 1.19 (0.94-1.51) | 1.04 (0.82-1.34) | 1.36 (1.09-1.71)** | 1.21 (0.95-1.54) | 1.29 (1.07-1.56)** | 1.14 (0.94-1.39) |
| Once a month or less | 1.27 (1.07-1.50)** | 1.21 (1.02-1.45)** | 0.99 (0.82-1.21) | 0.95 (0.77-1.16) | 1.11 (0.95-1.30) | 1.05 (0.89-1.23) |
| Refused | 1.36 (1.12-1.65)** | 1.29 (0.95-1.76) | 1.21 (0.95-1.54) | 1.04 (0.81-1.35) | 1.31 (1.09-1.58)** | 1.30 (0.91-1.84) |
| **As far as you know what your partner’s HIV status is?** |  |  |  |  |  |  |
| Negative | Ref | Ref | Ref | Ref | Ref | Ref |
| Positive | 0.64 (0.49-0.85)** | 0.66 (0.41-1.05) | 0.36 (0.25-0.51)*** | 0.34 (0.17-0.66)** | 0.45 (0.34-0.59)*** | 0.42 (0.25-0.69)** |
| Unknown | 0.74 (0.62-0.90)** | 0.79 (0.58-1.06) | 0.63 (0.51-0.77)*** | 0.74 (0.53-1.04) | 0.68 (0.57-0.80)*** | 0.73 (0.55-0.95)** |
| **HIV serostatus concordance** |  |  |  |  |  |  |
| Both negative | Ref | Ref | Ref | Ref | Ref | Ref |
| Both positive | 0.63 (0.43-0.94)** | 1.13 (0.58-2.21) | 0.32 (0.20-0.51)*** | 1.21 (0.53-2.74) | 0.44 (0.31-0.63)*** | 1.32 (0.70-2.51) |
| HIV serodiscordant/ unknown status | 0.87 (0.71-1.09) | 1.08 (0.79-1.49) | 0.67 (0.52-0.87)** | 0.93 (0.67-1.29) | 0.77 (0.64-0.94)** | 1.04 (0.79-1.36) |
| Both unknown | 0.48 (0.34-0.68)*** | 0.66 (0.40-1.08) | 0.39 (0.27-0.56)*** | 0.60 (0.36-0.99)** | 0.46 (0.34-0.62)*** | 0.70 (0.45-1.08) |
| **Network size** |  |  |  |  |  |  |
| Smaller | Ref | Ref | Ref | Ref | Ref | Ref |
| Larger | 1.46 (1.14-1.87)** | 1.50 (1.16-1.93) | 1.45 (1.13-1.87)** | 1.54 (1.19-2.00)** | 1.50 (1.21-1.85)*** | 1.56 (1.26-1.93)*** |
| Unknown | 1.09 (0.76-1.56) | 1.22 (0.82-1.81) | 1.12 (0.77-1.62) | 1.26 (0.83-1.90) | 1.24 (0.92-1.66) | 1.33 (0.96-1.83) |
